# Feedback parameters for a closed-loop multiple-input multiple-output model of the upper limb

**DOI:** 10.1371/journal.pcbi.1013183

**Published:** 2025-06-30

**Authors:** Ian Syndergaard, Daniel B. Free, Dario Farina, Steven K. Charles

**Affiliations:** 1 Mechanical Engineering, Brigham Young University, Provo, Utah, United States of America; 2 Bioengineering, Imperial College London, London, United Kingdom; 3 Neuroscience, Brigham Young University, Provo, Utah, United States of America; Universite Catholique de Louvain, BELGIUM

## Abstract

Both closed-loop models and multi-input multi-output (MIMO) models of the neuromusculoskeletal system of the upper limb are important for simulating and understanding motor control. Yet no large-scale linear neuromusculoskeletal models of the upper limb that are both closed-loop and MIMO have been developed. The primary difficulty in creating such models is choosing appropriate feedback parameters (such as feedback gains and delays), as such a collection of parameters is not available in the literature. The purpose of this work is to 1) present a method for developing MIMO models of short-loop afferent feedback and 2) offer estimates of average feedback parameter values and ranges based on the currently available literature. To this end, we combined measurements of feedback-related parameters available in 26 prior studies with known properties of system stability and behavior. As a result, we present estimated feedback gains and delays for a linear model of the upper limb with inputs into the 13 major superficial muscles and outputs to the 7 main joint degrees of freedom from the shoulder to the wrist. This model includes homonymous feedback mediated by Golgi tendon organs and both homonymous and heteronymous feedback mediated by muscle spindles. As a partial validation of muscle-spindle feedback gains, we compared the sign of the estimated gains to known differences in excess central delay between excitatory and inhibitory connections. The comparison proved correct in all 39 muscle pairs for which we had both estimated a feedback gain and found a measured excess central delay value in the literature. Furthermore, as a partial validation of delay times, we compared estimated delay times to measured innervation lengths. We found a strong fit for efferent delays (R = 0.88) and a moderate fit for afferent delays (R = 0.65). In addition, we demonstrate the effect of feedback on model behavior and present brief comparisons between this behavior and experimentally observed behaviors of the human upper limb with and without feedback.

## Author summary

Computational modeling is an essential tool for analyzing upper-limb behavior; yet representing neural feedback in a large-scale model of the upper limb has proven very challenging. When considering neural feedback connections between n muscles, there are $n^{2}$ possible connections to consider for each feedback mechanism, and modeling each feedback connection may require multiple feedback parameters (such as gain and delay values). A collection of feedback gains and propagation delays for each of these connections is not yet available in the literature. The purpose of this work is to 1) present a method for developing large-scale linear models of the upper-limb that include neural feedback and 2) offer estimates of average feedback parameter values and ranges based on the currently available literature. To accomplish these objectives, feedback-related parameters measured in 26 prior studies were combined with known properties of neuromusculoskeletal system behavior, resulting in a collection of parameter estimates for a linear model of the upper limb that includes the 13 major superficial muscles and the 7 main joint degrees of freedom from the shoulder to the wrist. Lastly, the simulated effects of neural feedback on system behavior were demonstrated.

## 1 Introduction

Computational models of the neuromusculoskeletal system of the upper limb have been instrumental in achieving a richer understanding of motor control, motor learning, movement disorders, and rehabilitation [1–6]. Such models often take neuromuscular signals or joint torques as inputs and predict joint or hand displacements as outputs (or vice versa) [7–15]. The simplest models are linear, single-input single-output (SISO), and open-loop (i.e., ignoring neural feedback mechanisms). Higher-fidelity models include multiple-input multiple-output (MIMO) models that take inputs to multiple muscles and predict outputs in multiple degrees of freedom (DOF) [11–13,16–19]. Other higher-fidelity models are closed-loop, capturing the effects of feedback mechanisms known to exist in the arm; the two feedback mechanisms most commonly modeled are short-loop afferents from muscle spindles and Golgi tendon organs (GTO) [7–10,20–22]. Both SISO closed-loop models and MIMO open-loop models have been developed and used to understand upper-limb motor control (references above); however, to our knowledge, no large-scale linear neuromusculoskeletal model of the upper limb that is both MIMO and closed-loop has been developed. Such a model could be an important tool for investigating the effect of neural feedback mechanisms in upper-limb motor control.

Creating neuromusculoskeletal models that are both MIMO and closed-loop requires MIMO models of the afferent feedback. Unfortunately, our lack of knowledge of feedback parameter values (such as feedback gains and delays) remains an impediment to creating such models. Prior SISO studies including feedback generally used simple linear time-invariant models of feedback, approximating the effect of feedback mechanisms using a proportional or proportional-derivative feedback loop. Some prior SISO closed-loop studies have estimated feedback parameters by optimizing the parameter values so that model predictions match experimental data [5,12,18,23]. Others have overcome uncertainty in feedback parameter values by simulating across a wide range of possible values [7,10,14]. While suitable for SISO studies, these approaches quickly become intractable for MIMO models with uncertainty in many feedback parameters. Other SISO studies have estimated feedback gains (or at least found limited ranges for these gains) through assumptions such as system stability [8,15,24]. Such assumptions can be extended to MIMO models and enable the determination of plausible ranges for feedback parameter values.

In addition, experimental studies have been conducted to measure some feedback parameters, such as the relative strengths of muscle spindle feedback connections between a number of upper-limb muscles. Similarly, the neural transmission delays associated with feedback have been measured between many upper-limb muscles. Combining these measured feedback parameter values with assumptions about system stability and simulations across a range of values of a small number of unknown parameters offers an opportunity for estimating feedback parameters throughout the upper limb, and thus the creation of a MIMO model of the upper limb including afferent feedback.

The purpose of this work is to 1) present a method for developing linear time-invariant MIMO models of short-loop afferent feedback and 2) offer our best estimates of average feedback parameter values and reasonable ranges based on the currently available literature. These estimates include feedback gains and delays for a MIMO model including muscle spindles and Golgi tendon organs in the 13 major superficial muscles of the upper limb. Although the true feedback gain values are unknown and vary with the state of the system, we provide partial validation of the sign (excitatory or inhibitory) and relative strengths of these gains. In addition, we present brief comparisons between the behavior of open- and closed-loop models of the upper-limb vs the behavior of the human upper limb with and without feedback. The feedback parameter values presented here can be used to develop closed-loop MIMO models of the neuromusculoskeletal system of the upper limb.

## 2 Results

### 2.1 Muscle spindle feedback gains

#### 2.1.1 Step 1: Extracting relative feedback gains from individual studies.

In the literature, we found information about relative muscle-spindle-mediated feedback connection strength for 70 of the 13^2^ = 169 muscle pairs (41.4%) examined in this study (Table 1). As the processing of these values required multiple steps, we included intermediate results in S3 Text. Values after the first processing step (dividing feedback connection strength between muscles when the stimulus was electrically applied to a nerve innervating multiple muscles at a site proximal to those muscles) can be seen in Table A in S3 Text. Intermediate values generated by averaging between repeated measurements in comparable studies are shown in Table B in S3 Text. This yields up to one intermediate value per muscle pair for each method. At this point, values from different methods still have different scaling and are therefore not yet comparable.

**Table 1:** Gain precursor values. The gain precursor from the $j^{th}$ source muscle to the $i^{th}$ target muscle, denoted as ${\tilde{G}}_{i,j}$, is assigned to the $i^{th}$ row and $j^{th}$ column of $\tilde{G}$. Values of the same color are on the same scale and can be compared to each other. References are indicated in superscripted brackets. Superscripted Greek letters indicate that the value must be divided between muscle pairs. Entries along the diagonal represent homonymous feedback [25–48].

Reflexes involving Bi and Tri as target muscles have been particularly well studied, with measurements from nearly all source muscles included in this study and considerable overlap between studies. In contrast, afferent connections to several target muscles remain sparsely studied; notably, none of the identified studies reported measurements of reflex activity involving the Lat Delt or Bra as target muscles, and measurements from only two or three source muscles were reported for reflexes involving Brd, PT, and ECU as target muscles. A large block of gains from proximal source muscles to distal target muscles (specifically, from all three deltoid heads and from Pec to Brd, PT, ECR, ECU, FCR, and FCU) remains blank because no quantitative information about these connections was described in the literature. Multiple authors [30,46,49] assert that short-loop reflex connections between proximal and distal muscles (such as those in this empty block) do not exist in the human upper limb (though they only examined excitatory feedback).

Qualitatively, the different methods used to quantify the relative strength of feedback connections agreed well. Differences in the sign of the gain (excitatory vs inhibitory) between methods were uncommon. In most cases, such differences in sign occurred when the feedback connection strengths were near zero; the only notable exception occurred between [41] and [46] and involved reflex loops from the wrist extensors to Bi and Tri.

#### 2.1.2 Step 2: Combining PSTH and EMG-A methods.

Because the relative feedback gains listed in Table 1 were measured using 7 different methods (represented as different colors in Table 1) with different scaling factors (and indeed different units), it was necessary to scale intermediates from each method. After generating the values found in Table B in S3 Text, the final intermediate step was implemented, as described in section 4.2.2. Each method shared at least one cell (i.e., one source-target pair) with the methods depicted in red or light blue. Therefore, values sharing a cell with light blue were scaled to the light blue scale, after which all values were scaled to the red scale as follows. By assuming that feedback gains calculated from two methods differed only by a scalar, a linear regression (passing through the origin) of the intermediates of two colors (one of which was light blue or red) in Table B in S3 Text was computed. The slope of the regression is the scalar value which, when applied to one of the methods, puts values of that method on the same scale as the light blue or red method, thus making intermediate values for these methods comparable. This was implemented for each method, resulting in the comparable intermediate values of Table C in S3 Text. Parameters for the regression of values from different measurement methods along with descriptive statistics are provided in Table D in S3 Text.

Quantitatively, the different methods also agreed well (see Table D in S3 Text). In *all* 7 *cases,* the regression coefficients were positive, meaning that the different methods agreed on the signs of the various feedback gains. Furthermore, for methods sharing intermediates for multiple muscle pairs, the correlations were strong (0.80 ≤ R ≤ 0.98) and statistically significant (at a 95% confidence level) except for the relationship between studies indicated in red and light blue (which still had a strong though less significant correlation coefficient: R = 0.82, p = 0.092).

After the scaled feedback connection strengths were averaged within each cell (to provide the final G matrix) we observed that the feedback gain values for more distal muscles tended to have larger magnitudes than those of more proximal muscles. This is consistent with anatomical findings from Gandevia, who notes that the density of afferent muscle spindle fibers is higher in distal muscles than in proximal ones [50]. In addition, we found the gain matrix to contain significant asymmetries: considering the 20 muscle pairs with connection strengths reported in both source-target directions, there was only moderate correlation between symmetric gains (R = 0.47, p = 0.038), and 5 of the 20 muscle pairs (Bi-PT, Bi-FCR, Bi-FCU, Tri-ECR, and Brd-PT) even exhibited differences in the sign of the gain (Table 2).

**Table 2:** The unscaled matrix of muscle spindle feedback gains, G.

		Target Muscle												
		Delt Ant	Delt Lat	Delt Post	Pec	Bi	Tri	Bra	Brd	PT	ECR	ECU	FCR	FCU
Source Muscle	Delt Ant	0.23				0.36								
	Delt Lat	0.23				0.31								
	Delt Post	0.23		0.50	-0.067	-0.003	0.013							
	Pec			-0.052	0.50	0.035	0.002							
	Bi	0.23		0.008	0.024	0.50	-0.19			-0.50	0		-0.22	-0.29
	Tri	0.41		0.005	0.005	-0.30	0.50				-0.28		-0.25	-0.31
	Bra					-0.36	-0.23							
	Brd			0.091		-0.42	-0.29			-0.55	0.39		-0.24	
	PT					0.43	-0.2		0.31		0.46			
	ECR	0.38		0.091		-0.058	0.19			0.47	1.24	0	-0.47	
	ECU			0.091		-0.058	0.19				1.24	0.96		
	FCR	0				0.32	-0.25		-0.15		-0.46		1.2	0.59
	FCU	0		0.41		0.19	-0.15						1.2	2.6

#### 2.1.3 Step 3: Scaling relative gains to absolute gains.

**Fig 1:**
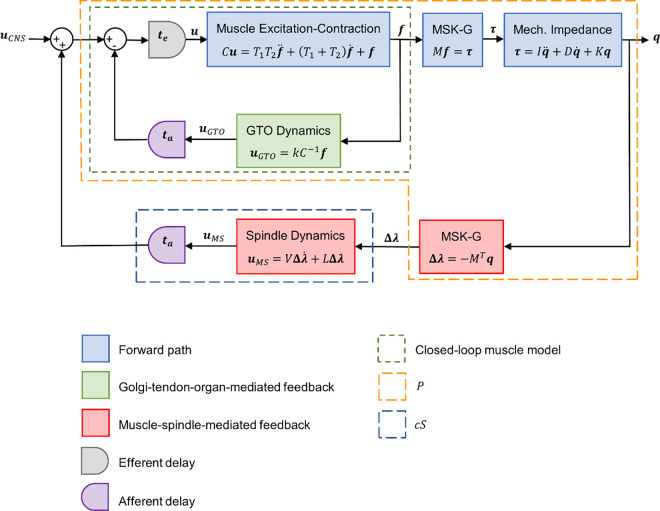
Closed-loop MIMO model of the upper limb. Feedback mediated by Golgi tendon organs (GTO) is modeled as force feedback, and feedback mediated by muscle spindles (MS) is modeled as proportional-derivative feedback. $u_{CNS}$: vector of neural drives from central nervous system to muscles; $t_{e}$ and $t_{a}$: vectors of efferent and afferent delays, respectively; C: diagonal matrix of maximum muscle forces; u and f: vectors of muscle activity and force; $T_{1}$ and $T_{2}$: diagonal matrices of muscle time constants; M: matrix of muscle moment arms representing the musculoskeletal geometry (MSK-G) of the upper limb; τ and q: vectors of muscle force and joint torque; I, D, and K: matrices of joint inertia, damping, and stiffness representing the mechanical impedance of the upper limb; Δλ: vector of muscle elongation; L and V: matrices of proportional and derivative feedback gains; $u_{MS}$ and $u_{GTO}$: vectors of neural outputs from MS and GTO, respectively; $kC^{-1}$: matrix representing the force feedback gain. P and cS represent subsystems referenced in Methods.

As described in Methods, matrix G (Table 2) must still be scaled by unknown factor c to obtain the matrices of derivative feedback gains (V=cG) and proportional feedback gains ($L=\frac{1}{r}cG$). An upper limit for c was found by performing a stability analysis on system PS (the computational model from descending neural drive to afferent signals, as depicted in Fig 1). As the gain had not been identified for the Delt Lat, but had been identified for the other heads of the same muscle, we assumed the unidentified gain to be the average of the identified gains. All other muscle spindle feedback gains that could not be estimated from the literature (blank cells in Table 2) were assumed to be zero. The stability analysis was performed on the entire system, including force feedback and delays, so we used the GTO gains and delay values described below. Other model parameter values are described by Davidson et al. and Corie et al. [16,17] (Tables A and B of S1 Text).

To perform the stability analysis, multiloop gain margins were calculated, and c was set to the upper limit of the gain margin (so that the system was on the verge of instability), which we termed the system-wide gain margin. This value was found to be 0.572, indicating that c lies in the range between 0 and 0.572 (incidentally, performing the stability analysis without force feedback yielded an upper limit of 1.0282). In summary, the matrices of derivative feedback gains (V) and proportional feedback gains (L) were calculated as V=cG and $L=\frac{1}{r}cG$, where 0≤c≤0.572, 0.06≤r≤0.14 (see Methods), and matrix G is given in Table 2.

To determine the sensitivity of the system-wide gain margin to changes in the parameter values of the forward path, the sensitivity analysis was repeated while halving and doubling the values of $T_{1}$, $T_{2}$, C, M, I, D, and K. The system-wide gain margin was found to be most sensitive (quadratically sensitive) to M; since M simply scales the model and appears in both the forward and feedback paths, halving and doubling M results in exactly four times and one fourth the gain margin, respectively (Table 3). Furthermore, the system-wide gain margin was linearly sensitive to C; since C also simply scales the model but only appears in the forward path, halving and doubling C resulted in exactly twice and half the gain margin, respectively. The sensitivity of the system-wide gain margin to all other parameters was less than linear, i.e., multiplying one of the other parameters by some value either multiplied or divided the gain margin by less than that value (Table 3).

**Table 3:** Sensitivity of the system-wide gain margin, defined as the maximum value of the muscle spindle gain scaling factor, c (before reaching instability). The values indicate the system-wide margin resulting from halving or doubling the default values of the indicated forward-path parameters.

Parameter	System-wide gain margin after scaling each parameter		
	Halved	Default	Doubled
T1 (muscle excitation time constant)	1.04	1.23	1.83
T2 (muscle contraction time constant)	0.89	1.23	2.11
C (vector of maximum muscle forces)	2.45	1.23	0.61
M (matrix of muscle moment arms)	4.90	1.23	0.31
I (matrix of joint inertia)	1.29	1.23	1.38
D (matrix of joint damping)	0.73	1.23	2.15
K (matrix of joint stiffness)	1.16	1.23	1.57

### 2.2 Golgi tendon organs

Using Eq. 7 with k=1.27 as recommended by Van der Helm and Rozendaal [8], we calculated GTO feedback values for each muscle (Table 4).

**Table 4:** The 13 major superficial muscles of the upper limb included in this study, with their associated abbreviations and maximum muscle force values from the literature, as well as our estimates of homonymous GTO feedback gains and afferent and efferent delays. Note that the GTO feedback gains are expressed in units of 10^-3^ MVC/N (e.g., the gain for Delt Ant is 1.04·10^-3^ MVC/N).

Muscle	Abbreviation	Maximum Muscle Force (N)	GTO Feedback Gain (10^–3^ MVC/N)	Afferent Delay (ms)	Efferent Delay (ms)
Anterior Deltoid	Delt Ant	1218.9	1.04	4.55	4.37
Posterior Deltoid	Delt Post	1103.5	1.15	4.55	4.37
Lateral Deltoid	Delt Lat	201.6	6.30	4.55	4.37
Pectoralis Major	Pec	658.3	1.93	4.57	8.05
Biceps	Bi	841.9	1.51	6.43	11.94
Triceps	Tri	1489.3	0.85	6.75	11.87
Brachialis	Bra	1177.4	1.08	6.20	11.13
Brachioradialis	Brd	276	4.60	6.09	13.63
Pronator Teres	PT	557.2	2.28	4.65	14.91
Extensor Carpi Radialis	ECR	407.9	2.15	8.89	16.98
Extensor Carpi Ulnaris	ECU	479.8	6.58	9.41	16.93
Flexor Carpi Radialis	FCR	589.8	3.11	9.25	14.95
Flexor Carpi Ulnaris	FCR	192.9	2.65	14.23	18.79

Because force feedback gains were assumed to be inversely proportional to maximum muscle force (see Methods), larger muscles were estimated to have lower feedback gains, while smaller muscles were estimated to have larger feedback gains. Since maximum muscle force tends to decrease proximally to distally, feedback values tended to increase from proximal to distal. For a typical 50^th^ percentile male, these gains ranged from $0.85\cdot 10^{-3}\frac{MVC}{N}$ (in the triceps) to $6.58\cdot 10^{-3}\frac{MVC}{N}$ (in ECU), with a mean of $2.85\cdot 10^{-3}\frac{MVC}{N}$ and a standard deviation of $1.78\cdot 10^{-3}\frac{MVC}{N}$.

### 2.3 Efferent, afferent, & central delays

Round-trip delay values and excess of central delay values were gleaned from the literature as described above. We found round-trip delay values for 63/169 muscle pairs (Table 5). For several muscle pairs (especially Bi, Tri, and wrist flexors and extensors), we found multiple values in the literature and averaged them to obtain a single value. Excess of central delay values were found for 43/169 muscle pairs (Table 6). Finally, we calculated afferent and efferent delays for all muscles (Table 4) using the least-squares pseudo-inverse method described in S2 Text.

**Table 5:** Round trip delay times (in milliseconds) reported in the literature. References are indicated in superscripted brackets [26–28,30–35,37–42,44,46,47,51,52].

**Table 6:** Excess of Central Delay values (in milliseconds) reported in the literature. Cells are color-coded according to the sign of the feedback gain estimated above in Table 2). Blue and red represent positive (excitatory) and negative (inhibitory) feedback gains, respectively. By definition, there is no excess of central delay for homonymous reflex loops (depicted in gray). White cells do not have a corresponding entry in Table 4.

		Target Muscle										
		Delt	Pec	Bi	Tri	Bra	Brd	PT	ECR	ECU	FCR	FCU
Source Muscle	Delt		1.5	0.067	0		0.1					
	Pec	1.5		0	0							
	Bi	0	0		1.22			1			1.5	1.5
	Tri	0	0	1.22					1.5		1.5	1.5
	Bra											
	Brd			0.95	1.5			1	0		1.05	
	PT			0	1.2		-0.07		0			
	ECR			1.2	0		0	0			0.817	
	ECU			1.2	0						0.95	1
	FCR			0	1.2		0.86		0.983	1		
	FCU										0	

### 2.4 Effect of feedback on system response

The open-loop system has 30 real and 14 complex poles, and the closed-loop system (represented using a third-order Padé approximation to include internal delays) has 21 real and 68 complex poles (Table 7 and Fig A in S4 Text). In a coupled multi-input multi-output system such as this, the poles are associated with the modes of the system rather than individual inputs or outputs. The inclusion of afferent feedback (with c at 75% of the stability limit) added clusters of conjugate poles around 15 ± 35i rad/s and -200 ± 60i rad/s, as well as a set of zeros and poles along the real axis (see Fig A in S4 Text, encircled in red, yellow, and green, respectively). Poles in red had a natural frequency, damping ratio, and time constant near 6.1 Hz, 0.39, and 67ms, respectively. For comparison, the poles in yellow have a natural frequency, damping ratio, and time constant near 33 Hz, 0.96, and 5ms, respectively, so their free-response oscillations die out quickly. The poles in green are overdamped, so their system modes are not associated with a natural frequency.

**Table 7:** Locations and characteristics of poles associated with the open-loop system and a third-order Padé approximation of the closed-loop system. Summary statistics of the imaginary portion of poles were calculated using the absolute value of the imaginary portion. In calculating summary statistics of natural frequency and damping ratio, real poles were excluded.

		Real (Hz)	Imaginary (Hz)	Time Constants (s)	Natural Freq. (Hz)	Damping Ratio
**Open-Loop**	**Mean**	-3.62	0.78	1.02	2.89	0.38
	**SD**	1.85	1.43	2.21	2.18	0.18
	**Min**	-5.31	0	0.19	0.67	0.15
	**Max**	-0.1	5.04	10.21	6.95	0.69
**Closed-Loop**	**Mean**	-19.14	5	0.63	21.13	0.67
	**SD**	20.77	3.88	1.57	19.76	0.32
	**Min**	-82.61	0	0.01	0.68	0.09
	**Max**	-0.1	11.09	9.82	82.61	1

To demonstrate the effect of feedback, we present the step responses in joint displacement caused by step inputs in descending neural drive. In addition, to compare with experiments, which more commonly involve a perturbation applied in external joint torque, we also present the step responses in muscle activity and joint displacement caused by step inputs in joint torque.

#### 2.4.1 Descending neural drive to joint displacement.

**Fig 2:**
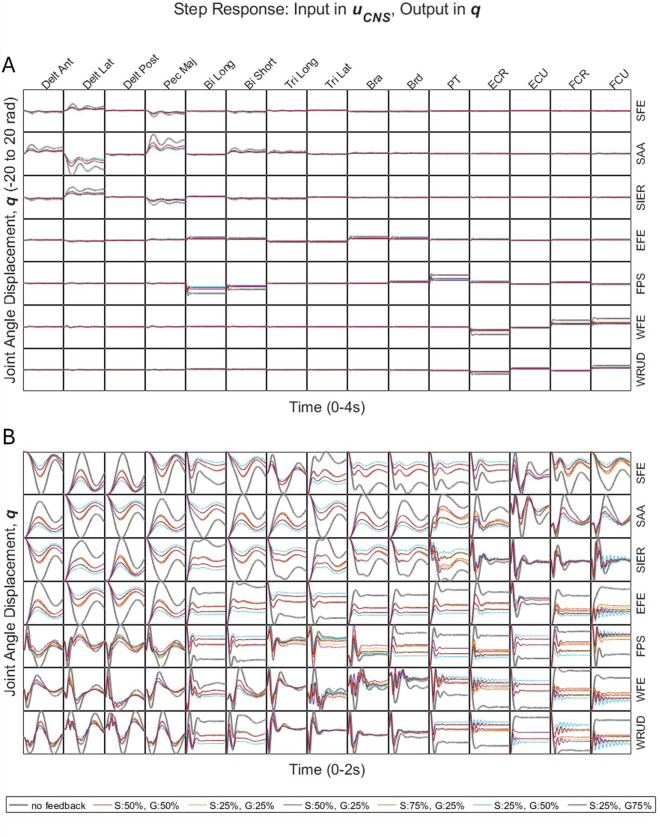
A) Step responses from descending neural drive to joint displacement (i.e., response in q due to step in $u_{CNS}$), with equal time and joint-displacement ranges (on the horizontal and vertical axes, respectively) for all subplots. The very large step responses in SAA (~20 rad, subplot A) are caused by large inputs (100% MVC), large maximum muscle forces and moment arms in Delt Lat and Pec Maj, and low joint stiffness in SAA. In actuality, tremorogenic inputs are on the order of 5-10% MVC, so responses would be scaled by 0.05-0.1, and joint stiffness increases significantly toward the ends of joint ranges of motion, further reducing motion amplitude. B) Same as A, but zoomed in, such that each subplot has its own distinct joint-displacement range (along the vertical axis).

A step input in descending neural drive to each of the 15 muscles produced a step response in each of the 7 DOF, for a total of 105 step responses (Fig 2). Because of interaction torques caused by the coupled impedance matrices, input to a given muscle produced a non-zero response in all DOF. Nevertheless, the largest responses occurred along the “diagonal” of this 7-by-15 matrix (Fig 2A), between muscles and the DOF on which they act directly (via muscle moment arms).

Combinations of GTO- and spindle-mediated feedback generally reduced steady-state error responses (Fig 2B). The percent overshoot, oscillation frequency, and settling time were relatively unaffected by feedback, except for step responses in distal DOF due to steps in distal muscles, where all three step-response characteristics (percent overshoot, oscillation frequency, and settling time) tended to increase. In these step responses in distal DOF due to step inputs to distal muscles, the highest level of GTO feedback included in the simulations (75% of the gain that produces instability) produced excessively underdamped responses, with numbers of oscillations far greater than those observed experimentally (see section 3.2.2). This suggests that the GTO gains in these distal muscle-DOF relationships may not reach such high levels, instead remaining further from the limit of instability.

We also considered the effect of feedback on the frequency response from descending neural drive to joint displacement (Fig B of S4 Text). In general, combinations of GTO- and spindle-mediated feedback tended to decrease the DC gain (magnitude ratio at 0 Hz) and add (or increase an existing) resonance peak around 1–6 Hz. The effect of feedback on the magnitude ratio fell off sharply above 8 Hz.

#### 2.4.2 External joint torque to muscle activity.

**Fig 3:**
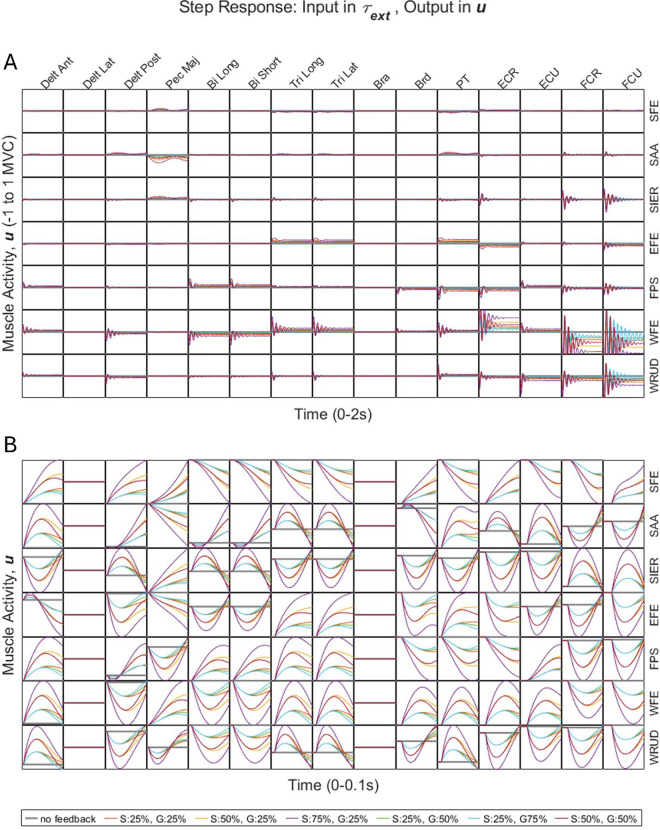
A) Step responses from external joint torque to muscle activity (i.e., response in u due to step in $\tau_{ext}$), with equal time and muscle-activity ranges (on the horizontal and vertical axes, respectively) for all subplots. B) Same as A, but zoomed in, such that each subplot has its own distinct muscle-activity displacement range (along the vertical axis).

Because the model is linear—and therefore does not include any non-linear elements to constrain muscle activity to be non-negative—the step responses in muscle activity included negative signals as well as positive signals (Fig 3A). The negative muscle activity, whose magnitude was small (the magnitude of the most negative signal was only 4% of MVC), should be interpreted as inhibition. In practice, researchers eliciting reflexes generally require some background activity (around 5% MVC or more) to make it possible to observe feedback-mediated inhibition.

To interpret the response in muscle activity in the context of this paper, we focused on the short-latency response (Fig 3B). As designed, delays in the responses increased with the distance of the perturbed muscle from the spinal cord. Without feedback, simulations exhibited no muscular response, as expected. With feedback, the magnitude of the response generally increased with feedback gains.

We were interested in whether lengthening of a muscle by a perturbation always resulted in excitation of that muscle and, similarly, whether shortening of a muscle always resulted in inhibition of that muscle. To test this, we focused on the first 25 ms (excluding delay) of the responses in muscle length change and muscle activity (Δλ and u in Fig 1) caused by a step perturbation in external joint torque. In 83% of cases, muscles were either unambiguously lengthened or shortened and unambiguously excited or inhibited. In over two-thirds of these cases (71 ± 10% (95% confidence interval)), perturbations in joint torque that lengthened a muscle resulted in excitation of that muscle, and perturbations in joint torque that shortened a muscle resulted in inhibition of that muscle. For example, an externally applied torque in wrist flexion lengthened the wrist extensors and shortened the wrist flexors, resulting in excitation of the wrist extensors and inhibition of the wrist flexors (Fig 3B). In the remaining cases, perturbations in joint torque that lengthened a muscle resulted in inhibition of that muscle (and vice versa for shortening). For example, an externally applied torque in wrist ulnar deviation lengthened the radial deviators (FCR and ECR) and shortened the ulnar deviators (FCU and ECU) but resulted in inhibition of all four muscles (Fig 3B). To understand this behavior of the model, it is important to keep in mind that whereas experimental measurements of stretch reflexes often involve only one DOF, our model has 7 DOF, none of which were constrained during perturbations. Consequently, a perturbation to any single DOF produces an effect (either lengthening or shortening) in *all* muscles. For example, in addition to lengthening the radial deviators and shortening the ulnar deviators, the externally applied torque in wrist ulnar deviation also lengthened the Delt Ant, Pec Maj, Tri Long, and Tri Lat muscles and shortened the Delt Lat, Delt Post, Bi Long, Bi Short, Bra, Brd, and PT muscles (Fig C in S4 Text). Because of heteronymous feedback, the response in muscle activity in a given muscle is a linear combination of changes in length (and rate of change of length) in many muscles (Table 2). Consequently, whether a lengthened muscle is excited depends also on the lengthening/shortening of other muscles as well as on the coefficients of the linear combination (feedback gains), so it is not surprising that the model predicts that lengthened muscles are sometimes inhibited and shortened muscles are sometimes excited. This phenomenon (of shortened muscles exhibiting a faciliatory response and lengthened muscles exhibiting an inhibitory response) has been observed experimentally [53].

#### 2.4.3 External joint torque to joint displacement.

**Fig 4:**
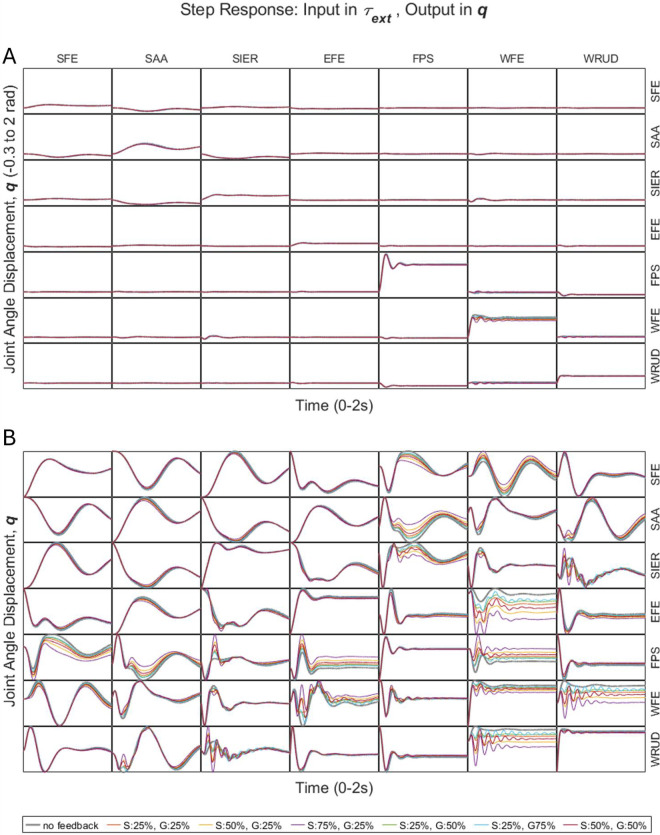
A) Step responses from external joint torque to joint displacement (i.e., response in q due to step in $\tau_{ext}$), with equal time and joint-displacement ranges (on the horizontal and vertical axes, respectively) for all subplots. B) Same as A, but zoomed in, such that each subplot has its own distinct joint-displacement range (along the vertical axis).

With and without feedback, the largest step responses generally occurred in input-output pairs involving the same DOF (along the diagonal in Fig 4A), and oscillation frequency generally increased proximal-distally (with regard to both input DOF and output DOF). Overall, feedback had a surprisingly small effect on most input-output pairs, including pairs involving the same DOF (diagonal cases in Fig 4B), where feedback reduced the steady-state response only slightly. In the few input-output pairs in which feedback had a large effect (e.g., step in WRUD torque and response in WFE displacement, or vice versa), feedback generally increased the percent overshoot and often increased oscillation frequency and settling time (especially in pairs involving distal DOF).

### 2.5 Partial validation

#### 2.5.1 Comparison of feedback gain sign to excess central delay.

Positive and negative feedback gains (Table 2) represent excitatory and inhibitory feedback, respectively. Similarly, excess central delay values near zero indicate excitatory feedback, whereas excess central delay values near 1–2ms indicate inhibitory feedback. Therefore, we corroborated the sign of our estimated feedback gains by comparing them to measured excess central delay values. The overlap was perfect: in all 39 source/target muscle pairs for which we had estimated a feedback gain and found a measured excess central delay value in the literature, positive feedback gains were associated with excess central delay values around zero, and negative feedback gains were associated with excess delay values around 1–2ms (Table 6). Thus, at least the sign of our feedback gain estimates are corroborated by values measured using a different neurophysiological measure.

#### 2.5.2 Comparison of delay times to innervation lengths.

**Fig 5:**
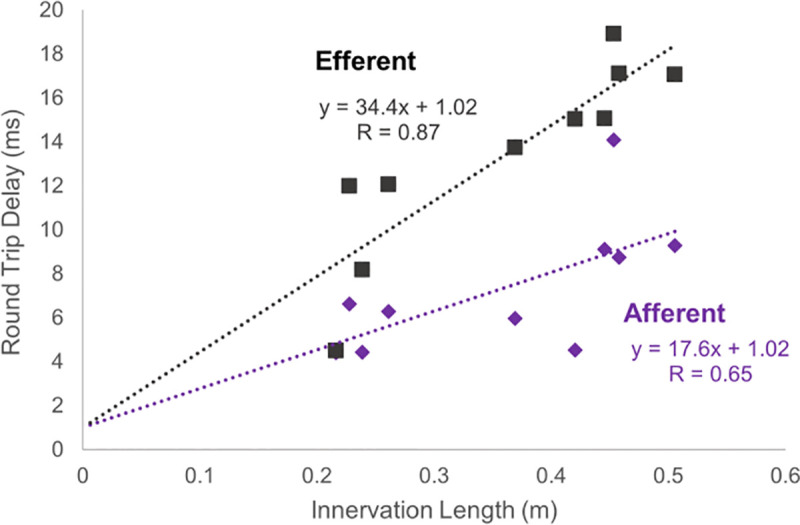
Round-trip neural delay times plotted against innervation length, together with the equations and correlation coefficients of their least-square line fits. The inverse of the slopes represent conduction velocities.

To corroborate our delay estimates, we regressed estimated delay values against innervation lengths [54] and found a strong fit for efferent delays (R = 0.88) and a moderate fit for afferent delays (R = 0.65), as depicted in Fig 5. The reciprocal of the slopes of the linear regressions are the average efferent and afferent neural conduction velocities: 29.1 m/s and 56.8 m/s for efferent and afferent conduction, respectively. These estimates are close to or within the range of conduction velocities for nerves of the leg available in the literature (believed to be similar to conduction velocities in the upper limb): 34–66 m/s for efferent conduction [55] and 12–74 m/s for afferent conduction [56,57]. The ratio of afferent to efferent conduction velocities (56.8/29.1 = 1.95) also falls nicely within the range of conduction velocity ratios available in the literature (1.6-2.0) [56,58,59].

#### 2.5.3 Comparison of feedback with estimated gains to feedback with pseudo-random gains.

To determine if the estimated feedback parameters are functionally advantageous compared to what would be expected by chance, we performed simulations using pseudo-random gains matrices. Specifically, we selected all elements of the matrix of proportional feedback gains (L) from a uniform distribution such that the absolute value of the randomly selected elements was less than or equal to the largest absolute value in the estimated version of L. For one set of simulations, the sign of the randomly selected value was constrained to be the same as its estimated counterpart. In the other set of simulations, we shuffled both the sign and value of each element. In all cases, system stability was required. Because the matrix of derivative feedback gains (V) is proportional to L (see section 4.2), all elements of V were pseudo-randomized as well. With the proportional and derivative feedback gains matrices thus pseudo-randomized (the GTO feedback gains remained unchanged), we simulated step responses in muscle activity caused by steps in external torque (as in 2.4.2). Through visual inspection of a large number of repeated simulations, we observed the following trends.

First, in simulated step responses using the estimated gains matrices, more than two-thirds of lengthened muscles were excited and shortened muscles were inhibited (section 2.4.2); this trend persisted for random gains matrices in which the sign of the elements was preserved, but it did not hold true for random gains matrices in which both values and signs were shuffled. Second, heteronymous short-term reflexes involving muscles located far from each other are known to be weak or absent (section 2.1.1); this behavior was observed in simulations with the estimated gains matrices, but not in simulations with the random gains matrices (whether or not sign were preserved). Third, simulated step responses using the estimated gains matrices were underdamped, and the dominant oscillation frequency tended to increase from proximal to distal (Fig 3A). More specifically, higher-frequency oscillations died out quickly in the simulated responses in proximal DOF but persisted in those of distal DOF. In contrast, there were no observable proximal-distal trends in the frequency of the dominant oscillations or how quickly higher-frequency oscillations died out, regardless of whether gain sign was preserved.

## 3 Discussion

In this paper, we have attempted to estimate—to the best of our ability based on prior studies—values of short-loop feedback gains and associated time delays needed for a linear, closed-loop, MIMO model of the human upper limb. This process required a number of assumptions that have been widely used to create feedback models in SISO systems (for a detailed discussion of the assumptions, see Limitations). Furthermore, the methods we employed could be implemented to create models of afferent feedback for other systems, such as the leg or neck.

### 3.1 Discussion of results

#### 3.1.1 Gain and delay values.

As this is the first attempt (as far as we are aware) to compile a list of short-loop feedback gain and delay values for muscles throughout the upper limb, we included partial validation of the muscle spindle feedback gain values and efferent, central, and afferent delay values.

Despite differences between different sources, the several methods used to quantitatively compare muscle spindle feedback agreed very well (R ≥ 0.80). Additionally, the sign of these feedback values agreed in all cases with the existence of inhibitory interneurons in the neural feedback circuit (as detected in the literature by measuring excess of central delay). As the source muscle was stretched, excitatory connections exhibited near-zero excess of central delay, while inhibitory connections always exhibited excess of central delay values of 1–3ms, indicating the presence of interneurons, as expected.

Delay values scaled proportionally with the length of the nerves innervating their respective muscles (R = 0.88 for efferent and R = 0.65 for afferent). Additionally, the inverse of this proportional scaling factor is the average conduction velocity, and these inverse values agreed with ranges for conduction velocity in the literature [56,58,59].

Unfortunately, we were not able to independently validate values for GTO feedback. Rather, the values presented in this paper depend on the validity of the assumptions made by van der Helm and Rozendaal [8]. Specifically, they assumed that Golgi tendon organ feedback is well-approximated by force feedback (a well-validated assumption [4,50,60–62]), the muscle/GTO complex must be stable (a logical assumption), and that the tradeoffs made between minimizing oscillation and increasing bandwidth are well-suited for all muscles of the upper limb. They claimed that their model can be broadly applied to muscles of the upper limb, but their validation only showed that their model predictions excellently matched measured forces and muscle activity in the shoulder and did not include measurements of muscle force and muscle activity in other upper-limb muscles.

#### 3.1.2 Effect of afferent feedback on system response.

Independent of the choice of input or output variable (descending neural drive to joint displacement, or joint torque to muscle activity or joint displacement), both open-loop and closed-loop systems were underdamped, with the dominant oscillation frequency of step responses increasing proximal-distally (except for the open-loop response in muscle activity, which was always zero). Considering responses in joint displacement caused by steps in descending neural drive or joint torque, we note the following trends. First, in both open- and closed-loop cases, the largest responses generally occurred in input-output pairs arranged along the “diagonal” in Figs 2 and 4. Specifically, in the transformation from descending neural drive to joint displacement (Fig 2A), the largest responses generally occurred between muscles and the DOF on which they act directly (via moment arms). Similarly, in the transformation from joint torque to joint displacement (Fig 4A), the largest responses generally occurred between torque and displacement in the same DOF. Second, feedback generally reduced the steady-state response, especially among “diagonal” input-output pairs (Figs 2B and 4B), though only very slightly for step inputs in joint torque. Third, the effect of feedback on the percent overshoot, oscillation frequency, and settling time of responses was generally smaller for pairs involving either proximal inputs *or* proximal outputs, and larger for pairs involving both distal inputs *and* distal outputs (Figs 2B and 4B). Fourth, high feedback gains (75% of values to reach instability) produced excessively underdamped responses in distal input-output pairs, suggesting that the gains of distal input-output pairs may remain further from the instability limit.

### 3.2 Comparison with prior studies

#### 3.2.1 Gain and delay values.

The idea of modeling afferent feedback is not new. In particular, a few notable studies have created nonlinear MIMO models of afferent feedback in the cat hindlimb which included both muscle spindle and GTO activity. These studies presented intricate models of short-loop afferent feedback including sophisticated models of muscle spindle and Golgi tendon organ activity [63–66]. Although their feedback parameters were generally estimated using optimization algorithms (rather than using direct measurements of neural activity as in this paper), their general trends agree well with the results of this paper. For both muscle spindles and GTO’s, homonymous feedback gains were found to be larger than heteronymous feedback gains; feedback values were generally smaller the farther from the diagonal of their respective matrices. Homonymous GTO feedback values reported by He et al. were robustly found to be negative [63], as modeled in our model. Whereas we were unable to find reliable prior measurements of heteronymous GTO feedback gains, some prior modeling studies included heteronymous GTO feedback gains, but they changed sign depending on the optimization parameters and task [18], which is incompatible with our linear time-invariant model. Nevertheless, simulations of GTO output from these models agreed with basic trends from our model: as forces increased, GTO outputs increased roughly linearly, at least when averaged and for small perturbations.

Although based on prior in-vivo measurements of reflex activity, the estimated gains and delays presented in this paper are novel and represent the largest collection of linear afferent feedback gains and delay values currently available in the literature. Nevertheless, it is not surprising that our estimates generally agree with the findings of the prior studies upon which our estimates are based.

Some prior studies went further and provided qualitative and limited quantitative analysis of a small number of muscle spindle connections in the arm. A notable exception is provided by Pierrot-Deseilligny et al., who tabulated the presence of excitatory feedback connections between many muscles of the arm, including qualitative comparisons between the strength of these feedback connections [49]. As in our study, Pierrot-Deseilligny et al. compiled studies of muscle spindle feedback from the literature [49]. Although not perfectly comparable (because of the lack of descriptions of inhibitory connections), our results agree well with the qualitative comparisons of excitatory connections they describe (as expected, since our sources included roughly two-thirds of the sources used by Pierrot-Deseilligny et al. [49]. Our work expands on the foundation they provided by including: 1) more muscles, 2) inhibitory feedback, 3) quantitative estimates of the strength of these feedback connections, and 4) delay values associated with these feedback paths.

This work also expands on SISO closed-loop models [8,67] and MIMO open-loop models [16,17] of the neuromusculoskeletal system. The development of these models was based on many of the same principles and assumptions used in this work and gave a starting point from which we could expand to include feedback from many muscles.

#### 3.2.2 Effect of afferent feedback on system response.

Comparing the model’s predicted behavior to previously observed experimental evidence was complicated by the model’s number of input-output relationships and simulated feedback levels (105 + 105 + 49 = 259 input-output relationships with 7 different feedback levels resulted in 1813 step response plots) and the large number of past studies investigating reflexes. In addition, past studies of reflex activity have typically isolated one or a small number of DOF by mechanically grounding the remaining DOF, whereas our model includes all 7 main DOF of the upper limb (from shoulder to wrist), allowing a perturbation in one DOF to lengthen/shorten all muscles, not just those spanning the perturbed DOF (Fig C in S4 Text). Consequently, the simulated step response of even a single input-output pair simultaneously exhibits a multitude of response characteristics (e.g., oscillation frequencies), and the definition or interpretation of other response characteristics (e.g., maximum overshoot or rise time) is ambiguous. Nevertheless, we compared the major features of observed reflex activity from two important studies to the major features of our simulated feedback activity. These two past studies were chosen to represent a range of experimental methods, observed features, and number of muscles and joint degrees of freedom.

***3.2.2.1 Comparison to experimentally observed single-DOF response with and without reflexes:*** We are aware of only one experimental study that measured the response of a joint to mechanical perturbations in vivo *with and without reflexes* [68]. Specifically, Sinkjaer and Hayashi measured the step response in wrist flexion-extension angle caused by steps in torque in that same DOF. Four subjects applied and maintained a small background torque of 0.1 Nm in wrist flexion. Then, the authors randomly applied a step perturbation of 1.8 Nm in wrist extension and measured the subjects’ subsequent wrist rotation. After obtaining control data, the authors repeated the perturbations without stretch reflexes, which they abolished by preventing blood flow to the forearm for about 20 minutes with the use of a blood pressure cuff.

We carefully compared the experimentally measured effect of the stretch reflex on the step response (in joint displacement caused by a step in joint torque) in a single DOF performed by Sinkjaer and Hayashi to the simulated effects of feedback in our multi-DOF model. Despite the difference in the number of DOF between their experiment (1 DOF) and our model (7 DOF), we found agreement on a number of fundamental behaviors and did not find any significant disagreements. First, Sinkjaer and Hayashi found that the actual neuromusculoskeletal system (between the torque perturbation and the resulting joint displacement) was remarkably linear. Specifically, the authors fit a linear, second-order impedance model (the single-DOF version of our third submodel) to their input and output data and found that the fit matched the experimental data within 5% for all four subjects, both with and without reflex. This provides partial justification for our use of a linear model. Second, Sinkjaer and Hayashi found the step responses with and without reflex to be underdamped, with damping ratios below 0.44 for all subjects. Similarly, both our open- and closed-loop systems included many complex poles (Fig A in S4 Text), with mean damping ratios of 0.38 and 0.67, respectively (Table 7). Third, Sinkjaer and Hayashi found that restoring the reflex decreased the damping ratio from a mean value of 0.36 without reflex to 0.21 with reflex. Similarly, we found that feedback often increased percent overshoot (which is inversely related to damping ratio), particularly in distal input-output pairs, including wrist flexion-extension (Fig 4B). Fourth, Sinkjaer and Hayashi found that restoring the reflex decreased the steady-state gain by about half. Similarly, we found that feedback decreased the steady-state response (though only slightly), particularly in WFE (Fig 4B). Fifth, Sinkjaer and Hayashi found that restoring the reflex increased the natural frequency ($\omega_{n}$) from 2.01 to 3.07 Hz and decreased the damping ratio (ζ) from 0.36 to 0.21; since the damped natural frequency of a linear second-order system is $\omega_{d}=\omega_{n}\sqrt{1-\zeta^{2}}$, the increase in natural frequency corresponds to an increase in oscillation frequency from 1.9 to 3.0 Hz. Similarly, we found that feedback increased the visually dominant oscillation frequency, particularly in distal DOF (Fig 4B). Sixth, Sinkjaer and Hayashi found that a step input in torque in wrist extension caused little change in wrist flexor activity without reflex but a large increase in flexor activity with reflex. Similarly, in our model, a step increase in wrist extension torque caused an increase in wrist flexor activity (see Fig 3B, which shows that a step input in wrist *flexion* torque causes a *decrease* in wrist flexor muscle activity, but since the system is linear, a step input in wrist *extension* torque would cause an *increase* in wrist flexor muscle activity).

***3.2.2.2 Comparison to experimentally observed multi-DOF response under different volitional conditions:*** We also compared our results to a recent sophisticated study of reflexes involving 8 muscles and all three rotational DOF of the shoulder [53], where agonist/antagonist relationships vary with the direction of volitional torque produced. For example, in shoulder abduction, the anterior and posterior deltoid muscles are agonists, but in horizontal flexion (rotating the arm in the horizontal plane), they are antagonists. In this study, Nicolozakes and colleagues tested the hypothesis that reflex activity depends not only on the direction of the perturbation, but also on the direction of volitional torque produced in the background. To test this hypothesis, the authors measured the amount of gain scaling, defined as an increase in reflex activity caused by an increase in the background activity of the muscle being stretched. They found that the gain scaling of short-loop reflexes indeed differed for different volitional torque directions, even when the kinematics of the perturbation (shoulder angle vs time) were identical. Thus, the stretch reflex in a given muscle can be modulated by the activity of other muscles.

The model presented here does not allow feedback gains to change with muscle activity. Our model is (deliberately) linear time-invariant, so its parameters do not change over time. This includes the gain matrices, precluding the possibility of scaling the feedback gains. Consequently, in our model, the spindle-mediated reflex (outer feedback loop in Fig 1) depends only on the kinematics of the perturbation (q in Fig 1), and not on background muscle activity. Thus, if there is no change in the amount that each muscle is lengthened/shortened by the perturbation, there cannot be a change in the simulated spindle-mediated reflex, even if the background activity of muscles changes (this is not to be confused with the model effect described in section 2.4.2, in which two perturbations producing the identical stretch in a given muscle can still produce different muscle-spindle-mediated reflexes in that muscle if the stretches produced in other muscles differ between the two perturbations). However, although this holds for the model’s spindle-mediated reflex, the model’s GTO-mediated reflex *does* depend on background muscle activity (since it depends on muscle force), so the *total* simulated reflex activity fed back to a muscle does depend on background muscle activity. In other words, the combination of spindle- and GTO-mediated feedback likely appears as some kind of gain scaling (in the sense that total reflex activity changes with background muscle activity) even though the actual feedback gains do not change. That said, although it has been speculated that involvement of force-sensitive Ib pathways could result in such complex stretch-reflex behavior [69], it is entirely unknown if the combination of spindle- and GTO-mediated feedback predicted by our model would reproduce the patterns of gain scaling observed experimentally.

### 3.3 Limitations

Despite the advancement that these results represent, they are certainly not without their limitations. Because relative feedback gains were not available in the literature for all source/target muscle pairs, the model lacks many feedback gain values (99/169 source/target muscle pairs). In order to use such a model in simulation, a value must be chosen. Multiple authors have asserted that trans-joint proximal-to-distal reflex connections do not exist (i.e., proximal to distal connections between muscles that do not span any of the same DOF, which includes 16 muscle pairs for the muscles included in this study), which would imply that many of these unknown values are zero [30,46,49]. Because even the sign of these unknown muscle feedback gain values is unknown, we suggest using a value of zero until further information is available. As more relative feedback gains become available in the literature, the matrix of afferent feedback gains should be updated.

To allow use of concepts and tools from linear systems theory to analyze system stability and capture system behavior, we deliberately modeled the forward path using simple submodels. For example, although the basic (i.e., linear) viscoelastic effects of muscle are included in the damping and stiffness matrices, the forward model does not include what are commonly known as the force-length and force-velocity relationships of muscle. Also, muscle moment arm and inertial values depend on posture, so these models and associated feedback parameters should only be used to model conditions in which assumptions of linearity and time-invariance are appropriate, such as static (or quasi-static) tasks.

The assumed structure of the feedback models (Golgi tendon organs are assumed to act as pure force feedback and muscle spindles are assumed to act as a PD controller, all with constant gains) is simplistic, ignoring both nonlinear and time-varying feedback-related phenomena. Feedback parameters are not invariant but can be modulated, e.g., by gamma motor neuron activity or Renshaw inhibition. Because gamma motor neuron and Renshaw activity can in turn depend on intent, level of muscle activation, displacement amplitude, and perturbation bandwidth [18,67,70,71], these feedback values can vary under different conditions. Indeed, gain values for a given muscle pair differed between sources in the literature, and occasionally even differed in sign. However, some confidence is gained by the statistically significant correlation observed between studies (R = 0.82, p = 0.020), after accounting for scaling between the different methodologies. Because feedback parameters can change under different experimental conditions, use of the parameter values presented in this paper should be limited to conditions similar to the experimental procedures from which these values were gleaned (static posture, modest background activity). Also, van der Helm et al. indicated that the constant of proportionality relating the proportional and derivative gains matrices (L and V) should lie within a relatively narrow range [8], but it is not yet clear whether that constant of proportionality is the same for all muscles (as was assumed for this paper). Despite these limitations, the assumption of linear time-invariant feedback mechanisms provides a good point of departure for future improvements.

The system-wide gain margin, defined as the upper limit for scaling the muscle spindle gains matrix, was based on a stability analysis that is dependent upon the forward-path model (in addition to the feedback-path model); different forward-path parameter values would yield a different scalar to apply to the feedback gains matrix. However, we analyzed the sensitivity of the system-wide gain margin to changes in forward-path parameters and found that the system-wide gain margin was most sensitive to muscle moment arms and maximum muscle force (M and C). Fortunately, the variability in these matrices is relatively straightforward (larger subjects tend to have greater muscle moment arms and maximum muscle forces), and their effects on the system-wide gain margin are easily understood (see Results). The parameters with the least predictable values are stiffness and damping (K and D), which can change significantly with muscle contraction. Fortunately, the system-wide gain margin is less sensitive to these parameters (Table 3). Lastly, feedback gain values are known to change under different conditions (such as during teeth clenching or the Jendrassik maneuver [72]). The variability in feedback gains has not been thoroughly evaluated or described in the literature, and the values presented in this paper only represent average variables taken across experimental conditions and subjects represented in the literature. Most often, experimenters measured relative magnitudes of gains (comparing between muscles). When multiple studies measured the same relative gains, they agreed reasonably well. We then used practical considerations from system dynamics and control to provide a scalar (and a reasonable range for this scalar) by which these relative gains must be scaled. In applying these considerations, we made a number of assumptions (see Methods). Thus, there is uncertainty in both the original measurements and in the assumptions used to derive further parameter values from the original measurements. Yet despite the uncertainty, MIMO models of feedback are an important tool and must start somewhere. The purpose of this study was to provide a first attempt at creating large-scale MIMO, closed-loop models of the neuromusculoskeletal system of the upper limb.

### 3.4 Conclusions

The aim of this study was to 1) present a method for developing linear MIMO models of short-loop afferent feedback and 2) offer our best estimates of feedback parameter values based on the currently available literature. To that end, we have completed a thorough review of models of afferent feedback and measurements of afferent connection strengths available in the literature to provide the most comprehensive collection of linear feedback parameters to date, as well as a model structure in which these parameters can be applied.

Simulation using MIMO models of the neuromusculoskeletal system remains a developing field. Rich models such as those used in OpenSim are refining our understanding of human movement and motor control and hold promise for optimizing treatments for individuals suffering from motor disorders. Nevertheless, it remains a challenge to incorporate models of feedback mechanisms which are known to exist in the neuromusculoskeletal system. Simple linear models of feedback are a natural first step. By including models of feedback such as that presented in this work, researchers can further enrich these models and continue to deepen our understanding of motor control.

## 4 Methods

### 4.1 Model structure

#### 4.1.1 Forward path.

As mentioned above, this paper focuses on developing a model of feedback that can be added to existing open-loop MIMO models to create closed-loop MIMO models of the upper limb. Although the method presented here can be used to expand many different open-loop models, the feedback parameter values estimated in this work were identified in part by a stability analysis involving a particular forward path. Therefore, we begin with a brief description of the forward path used in this study; for more details, see [16,17].

To produce motion of the upper limb, the central nervous system sends descending neural signals to upper-limb muscles. Through a cascade of processes (such as those depicted in the forward path of Fig 1), these neural signals are transformed into joint rotations in the 7 DOF of the arm from shoulder to wrist (listed in Table 8). Assuming that changes in the joint angles are small (such as when holding a posture or experiencing small perturbations), this cascade of transformations can be modeled as a simple linear, time-invariant system. In the first submodel, the set of descending neural signals $u_{CNS}$ (n×1 vector containing the time-varying signal to each of the n muscles included in the model) is transformed into muscle forces f (n×1 vector) according to their excitation-contraction dynamics approximated by a Hill-type muscle model with muscle time constants $T_{1}$ and $T_{2}$ and maximum contractile force C (listed in Table 4). All three ($T_{1}$, $T_{2}$, and C) are diagonal n×n matrices. By including as many inputs as muscles, the model is capable of full independent control of each muscle. In practice, many tasks are likely implemented using a smaller number of control signals [73,74], and such synergies could be implemented as constraints in our more general model. In the second submodel, the muscle forces act via moment arms M (m×n matrix equal to the transpose of the Jacobian from muscle space to joint space, where m is the number of joint DOF included in the model) to produce joint torques τ (m×1 vector). Values for M are listed in Table A in S1 Text. Finally, in the third submodel, the joint torques overcome the mechanical impedance of the limb (representing a combination of the inertial, damping, and stiffness properties of the limb, denoted by m×m matrices I, D, and K, respectively) to produce a change in joint angles q (m×1 vector). Values for M and  I are dependent upon the posture of the upper limb, which was set to be the default posture used in prior modeling studies (Posture 1 in [16,75]): 0 deg of shoulder flexion-extension, abduction-adduction, and internal-external rotation; 90 deg of elbow flexion-extension and forearm pronation-supination; 0 deg of wrist flexion-extension and radial-ulnar deviation. This places the entire upper limb in a parasagittal plane, with the upper arm also in the coronal plane and the forearm and hand also in the transverse plane, causing the back of the hand to face laterally and the thumb to point upward. Values for I, D, and K are listed in Table B in S1 Text. All three impedance matrices include off-diagonal (coupling) terms, which result in interaction torques. Note that since the damping and stiffness matrix values were gathered from past experimental measurements, in which joints were perturbed and the resulting responses were recorded, they include the basic (i.e., linear) viscoelastic effects of all perturbed tissues. That said, they do not include what are commonly known as the force-length and force-velocity relationships of muscle, which capture the change in force-generating capacity caused by activating a muscle at a different length or rate of change of length. Values for I, D, and K were taken from the literature [76–79] and are described in detail in [16,17], which included 15 muscles in their model [16,17]. Here, we considered the same muscles but grouped the heads of the biceps into a single muscle and the heads of the triceps into a single muscle, resulting in the 13 muscles listed in Table 4. Additional details of the forward path model can be found in S1 Text.

**Table 8:** The 7 main degrees of freedom of the upper limb from shoulder to wrist, with their abbreviations.

DOF	Abbreviation
Shoulder Flexion-Extension	SFE
Shoulder Abduction-Adduction	SAA
Shoulder Internal-External Rotation	SIER
Elbow Flexion-Extension	EFE
Forearm Pronation-Supination	FPS
Wrist Flexion-Extension	WFE
Wrist Radial-Ulnar Deviation	WRUD

#### 4.1.2 Feedback path.

We have included two primary feedback paths: short-loop reflex arcs involving afferents from type Ia muscle spindles and short-loop reflex arcs involving afferents from Golgi tendon organs (Fig 1). The details are described below, but we provide an overview here: in a short-loop reflex arc, muscle spindles or Golgi tendon organs in a *source muscle* generate an afferent neural signal in response to a change in that muscle’s state. This signal travels from the source muscle to the spinal cord, which involves an afferent time delay $t_{a}$ (n×1 vector). In the spinal cord, the feedback signal passes through one or more synapses (associated with a central time delay $t_{c}$, also an n×1 vector) and combines with descending signals. This composite signal then travels to a *target muscle*, which can be excited or inhibited by the signal originating in the source muscle. The transmission from spinal cord to target muscle involves an efferent time delay $t_{e}$ (n×1 vector).

The source muscle and the target muscle can be the same (homonymous reflex) or different (heteronymous reflex), and in general each source muscle may send neural signals to several target muscles [80]. Thus, for a model with n muscles, there are n×n potential feedback paths involving muscle spindles and n×n potential feedback paths involving Golgi tendon organs. Each GTO feedback path involves one feedback gain, and each muscle-spindle path involves one proportional feedback gain and one derivative feedback gain (more details below). Therefore, we seek three n×n matrices of feedback gain values, where the entry in the $i^{th}$ row and $j^{th}$ column represents the feedback gain value from the $j^{th}$ source muscle to the $i^{th}$ target muscle. We also seek the n×1 vectors of afferent and efferent time delays, $t_{a}$ and $t_{e}$.

### 4.2 Muscle spindle feedback gains

Muscle spindles are stretch receptors located within the belly of a source muscle. These receptors produce neural signals that travel to the spinal cord via type Ia and type II sensory fibers. Type Ia fibers carry information related to both the muscle’s length and time rate of change of length, so feedback relayed via these fibers is commonly modeled using a proportional-derivative (PD) controller [4,8,14,18,67,70,71,81]. Type II fibers carry information related to the muscle’s length, so models of feedback that include both type Ia and type II fibers often group both types into a single PD controller [8,14,70,81]. However, type II fibers conduct roughly half as fast as type Ia fibers [82], and since we determined plausible ranges for gain values using a stability analysis with delays, it was inappropriate to combine type Ia and type II feedback as a single PD controller. Instead, we focused on modeling type Ia feedback and ignored type II feedback.

Because each source muscle has the potential to send afferent feedback to each target muscle, a model containing PD feedback for n interconnected muscles requires an n×n matrix L of proportional feedback gains (with units of activation per unit length change) and an n×n matrix V of derivative feedback gains (with units of activation per unit length change per second). This simple linear model ignores mechanisms that modulate the feedback gains in response to changes in system state (such as posture, intent, fatigue, or level of contraction).

As suggested by van der Helm and Rozendaal, we assumed the structure of L and V to be the same; that is, we assumed that they differ only by a scalar proportionality constant: V=r L, where r has units of seconds [8,20]. Van der Helm and Rozendaal justified this assumption by stating that although “the length and velocity feedback gains … can vary over quite a large range, … their mutual relation remains between quite fixed boundaries,” which they gave as 0.06s<r<0.14s. This was determined in simulation by noting that joint stiffness depended on proportional feedback and joint damping depended on the ratio between derivative and proportional feedback, and that only a limited range of r values modeled realistic joint dynamics [20]. Van der Helm and Rozendaal suggested using the average value of r=0.1, which was also used independently by Winters and Stark [24]. Furthermore, van der Helm and Rozendaal showed that simulations using a model based on this assumption closely matched experimental data from Chen and Poppele [83]. Under this assumption, we only need to find one matrix, G, from which V and L can be determined, as described below.

Absolute values of the elements of V and L have not been described in the literature. However, the *relative* strengths of many type Ia feedback connections have been investigated in various prior studies. As a hypothetical example, a study may have shown that stimulating source muscle A caused twice as much reflex activity in target muscle B than in target muscle C. Thus, the reflex connection from A to B is twice as strong as the reflex connection from A to C. Assuming the feedback gains to be proportional to the strength of the reflex connections, the feedback gain from muscle A to B is twice as large as the feedback gain from muscle A to C. If the relative feedback connection strength from source muscle A to target muscle B is denoted by $G_{B,A}$ (the element in column A and row B of matrix G), then for this hypothetical example, $G_{B,A}=2G_{C,A}$.

Unfortunately, different studies used various techniques to elicit spinal afferents, including tendon taps, percutaneous electrical nerve stimulation, and electrical muscle stimulation. Similarly, different studies used a variety of techniques to measure the resulting response in the target muscle, including electromyography averaging and post-stimulus time histograms. These different techniques often resulted in estimates of feedback connection strength with different units, making it difficult to compare values from different studies. Instead, we used an indirect method that takes advantage of overlap between studies, following a three-step process. First, from a given study, we determined the relative feedback strength from source muscle j to target muscle i, denoted as ${\tilde{G}}_{i,j}$ (the tilde indicates that it is not yet compatible with other studies). Second, taking advantage of the overlap between studies, we scaled the gains ${\tilde{G}}_{i,j}$ from different studies to normalize them and make their scaling uniform, yielding a unitless matrix G of compatible *relative* feedback connection strengths. Third, the matrices of derivative and proportional feedback gains were calculated as V=cG and $L=\frac{c}{r}G$, where c is a constant with the same units as V. These steps are described in detail below.

#### 4.2.1 Step 1: Extracting relative feedback gains from individual studies.

Although the details of the individual experimental procedures differed, the measurement techniques generally followed one of two paradigms: post-stimulus time histogram (PSTH) or electromyogram averaging (EMG-A). We found 26 studies (see references in Table 1) that used six variations of these techniques to quantitatively evaluate feedback connections between many pairs of muscles. The strength of feedback connections reported by studies using the same variation were assembled into groups that can be compared directly to each other. In contrast, feedback connection strengths reported by studies using different variations have incompatible units and had to be scaled to make them compatible (see Step 2).

Studies applied a perturbation to a source muscle or nerve and observed the response in a target muscle; by comparing the magnitude of the response to the magnitude of the perturbation, a feedback connection strength can be estimated. Muscle responses typically occur in several phases, with the fastest mechanisms occurring first and slower mechanisms appearing later in the response. Because we focused on feedback from Ia afferents, we focused on the first phase of the response.

During these experiments, the subjects were seated at a table with the upper arm hanging downward and the forearm resting on the table surface. The hand was either pronated or supinated to allow EMG electrodes to be placed over the bellies of the muscles of interest. This posture is roughly similar to the default posture used to estimate muscle moment arms and inertial parameters in the model.

***4.2.1.1 PSTH methods:*** In PSTH studies, a target muscle was contracted to 5–10% of maximum voluntary contraction (MVC), where MVC was determined by first having the subject contract the muscle as much as possible against an immobile stop and measuring the resulting EMG response, and then marking the target EMG level (e.g., 5% MVC) and instructing the subject to maintain EMG at that level. A source muscle was then stimulated electrically or mechanically via a tendon tap, or a nerve innervating (a) source muscle(s) was stimulated electrically (generally just below motor threshold, MT, e.g., at 0.95·MT). A sample of individual motor units of the target muscle were observed through fine wire EMG in the target muscle’s belly, and their firing probabilities were compared to baseline (i.e., without stimulation) using a $\chi^{2}$ test. The number of observed motor units for which firing probability increased ($n_{+}$), for which firing probability decreased ($n_{-}$), and the total number of motor units observed ($n_{tot}$, some of which may not have changed firing probability) were reported. Assuming that, on average, excitation and inhibition in a motoneuron have equal strength (but opposite sign), we defined the relative PSTH feedback connection strength from source muscle j to target muscle i investigated in a given study as


$$\begin{array}{c} {\tilde{G}}_{i,j}=\frac{n_{+}-n_{-}}{n_{tot}} \end{array}$$ (1)

Feedback strength values for PSTH methods, by the above definition, must be between −1 and 1.

For some muscle pairs, the PSTH feedback connection strength was reported in multiple comparable studies; in this case, we took the average of the reported feedback connection strengths. If stimulation was applied to a nerve innervating multiple muscles and at a site proximal to these muscles, the observed feedback connection strength may represent feedback to a target muscle from multiple source muscles. In this case, $\frac{n_{+}-n_{-}}{n_{tot}}$ was assumed to be the sum of the feedback connection strengths from those muscles, and its value was distributed between the associated feedback connection strengths. For example, if a nerve innervating both muscles j and k was stimulated proximal to both j and k, then the connection strengths between these source muscles and target muscle i was defined as $\frac{n_{+}-n_{-}}{n_{tot}}={\tilde{G}}_{i,j}+{\tilde{G}}_{i,k}$. If ${\tilde{G}}_{i,j}$ was available by itself (e.g., from another study), then ${\tilde{G}}_{i,k}$ was obtained as ${\tilde{G}}_{i,k}=\frac{n_{+}-n_{-}}{n_{tot}}-{\tilde{G}}_{i,j}$. If neither ${\tilde{G}}_{i,\;j}$ nor ${\tilde{G}}_{i,k}$ were available by themselves, they were assumed to contribute equally, i.e., ${\tilde{G}}_{i,j}={\tilde{G}}_{i,k}=\frac{\left(\frac{n_{+}-n_{-}}{n_{tot}}\right)}{2}$.

***4.2.1.2 EMG-A methods:*** In EMG-A studies, a target muscle i was voluntarily contracted at a fraction of MVC or electrically stimulated to a fraction of MVC (determined through static contraction). This background activity $b_{i}$ was usually 0.05-0.10 (i.e., 5–10% of MVC) and displayed as EMG on an oscilloscope so the subject could try to hit and maintain a designated level. Then, a source muscle was stimulated electrically or mechanically via a tendon tap, or a nerve innervating a source muscle was stimulated electrically at a fraction of its motor threshold. The stimulation intensity in source muscle j was denoted by $s_{j}$ (generally around 0.9, i.e., just below motor threshold). Following stimulation of the source muscle, afferent feedback adds to the background activity of the target muscle. The average muscle activity of the target muscle was observed via surface EMG before stimulation ($b_{i}$) and after stimulation ($u_{i}$), and the fraction (p) by which the average activity increased following the stimulus was reported (decreases in activity are indicated by negative values of p).

The change in average activity in the $i^{th}$ target muscle depends on the magnitude of both its background activity ($b_{i}$) and the stimulation intensity in the $j^{th}$ source muscle ($s_{j}$). Because different studies used slightly different levels of background contraction and stimulation intensities, we accounted for these differences when calculating the strengths of the reflex connections. Prior studies have shown that a larger stimulus results in larger feedback signals [25,41,46,52]. We have, therefore, assumed that the afferent signal is proportional to the intensity of the stimulus, where the constant of proportionality (${\tilde{G}}_{i,j}$) may differ for each target-source muscle pair. Put succinctly,


$$\begin{array}{c} u_{i}=\;b_{i}+{\tilde{G}}_{i,j}s_{j} \end{array}$$ (2)

where ($u_{i}$) is the activity in target muscle i and is the sum of the background activity and afferent signal. $s_{j}$ is the stimulus in source muscle j which is scaled by ${\tilde{G}}_{i,j}$ to create the afferent signal.

The increase in muscle activity (expressed as a fraction) in target muscle i is


$$\begin{array}{c} p_{i}=\frac{u_{i}-b_{i}}{b_{i}} \end{array}$$ (3)

Substituting Eq. 2 into Eq. 3 yields


$$\begin{array}{c} p_{i}=\frac{b_{i}+{\tilde{G}}_{i,j}s_{j}-b_{i}}{b_{i}}=\frac{{\tilde{G}}_{i,j}s_{j}}{b_{i}} \end{array}$$ (4)

and thus, the strength of the reflex connection ${\tilde{G}}_{i,j}$ was calculated as


$$\begin{array}{c} {\tilde{G}}_{i,j}=p_{i}\frac{b_{i}}{s_{j}} \end{array}$$ (5)

Unlike PSTH methods, reflex connection strengths from EMG averaging methods are not restricted to the range from -1 to 1.

Similar to PSTH methods, when more than one reflex strength for a given source/target muscle pair was available, they were averaged. If the stimulation was applied to a nerve innervating multiple muscles and at a site proximal to those muscles, the percent increase was assumed to be the sum of the reflex strengths from those muscles, and its value was distributed between the associated reflex strengths, as explained above.

***4.2.1.3 Other technique variations:*** While all studies included here used some variation of PSTH or EMG-A methods, some studies reported values in a way that precluded creating estimates of feedback strength that were directly comparable to those from other studies. For example, values from studies using electrical stimulation could not be directly compared to values from studies using mechanical stimulation (tendon tap). These reflex strengths were calculated in a similar manner to the preceding sections (but were not combined with values from other studies until Step 2).

#### 4.2.2 Step 2: Combining PSTH and EMG-A methods.

To place the ${\tilde{G}}_{i,j}$ values obtained by PSTH and EMG-A methods on equal footing, we used source/target muscle pairs for which both PSTH and EMG-A estimates were available. We assumed feedback gains calculated from the two methods differed only by a scalar and calculated a linear regression passing through the origin. All EMG-A values were multiplied by the slope of the regression to make them comparable to PSTH values. This process was repeated for other methods that were not directly comparable (all methods shared at least one value with at least one other method). Finally, values from all methods were compiled into one matrix,  G, of compatible relative gains. Where multiple methods gave values for the same entry in G, the mean was taken.

#### 4.2.3 Step 3: Scaling relative gains to obtain absolute gains.

Because the reflex connection strengths in the relative gains matrix, G, obtained from past studies were only relative, they must be scaled to obtain an estimate of the absolute muscle spindle feedback gains. This scaling factor was estimated through a stability analysis of the closed-loop system. Stable systems under feedback control become unstable when feedback gains become too large. Stability is a common requirement in modeling studies, as typical human movements do not exhibit characteristics of unstable systems. By finding the threshold at which instability occurs, we can assert an upper limit for the feedback gain scalar (for additional details, see descriptions by van der Helm and Rozendaal [8] and Hespanha [84]).

Such a stability analysis requires a closed-loop model of the arm, such as that shown in Fig 1. For the forward path, we used the open-loop model described above. The inner feedback path represents Golgi tendon organ feedback (discussed below). The outer feedback path models muscle spindle feedback.

For a system to be stable, the loop-at-a-time stability criterion [85,86] requires that


$$\begin{array}{c} real(1-cS_{i}P_{k})<0 \end{array}$$ (6)

for all i and k, where $P_{k}$ is the forward path (plant) transfer function from the $k^{th}$ input muscle and ${cS}_{i}$ is the muscle-spindle feedback path transfer function for the $i^{th}$ target muscle, where c is the gain threshold applied to the outer loop before the closed-loop system becomes unstable. Because the system is MIMO, S and P are both matrices of transfer functions. $S_{i}$ is the $i^{th}$ row of S and $P_{k}$ is the $k^{th}$ column of P; thus $cS_{i}P_{k}$ is the afferent signal entering target muscle i due to descending neural commands in muscle k. The term $cS_{i}P_{k}$ is subtracted from one (as opposed to added) because the outer loop was formulated as positive feedback. In other words, for stability, all roots of the closed-loop model must be in the left half plane [84]. There are many values of c for which the system is stable; the value of c for which ${\left\|I-cPS\right\|}_{\infty }=0$ is the upper limit for c.

Because the system is MIMO, loop interactions must be considered. We found the upper limit of c using Matlab’s *diskmargin* function to calculate the system-wide multiloop gain margin, which accounts for loop interactions. Note that this system-wide gain margin would put the system on the verge of instability. In reality, the nervous system is likely to maintain a safety margin [8]. Of course, the value obtained for the system-wide gain margin depends not only on the values of the feedback parameters, but also on the parameter values used in the forward path. To evaluate the robustness of the system-wide gain margin to changes in the forward path, we repeated the sensitivity analysis while doubling and halving the values of the following forward-path parameters: $T_{1}$, $T_{2}$, C, M, I, D, and K (all elements of a matrix were either halved or doubled).

### 4.3 Golgi tendon organ feedback gains

Golgi tendon organs are sensory receptors located within the tendon of a muscle. They produce afferent signals in approximate proportion to the force in the muscle [50,60–62]; thus, their activity is commonly modeled as force feedback [4,8,18]. We note that while the firing rate of a single Golgi tendon organ is nonlinear and not well correlated with the tension in the muscle, the summed responses of an ensemble of Golgi tendon organs have been shown to vary linearly with force at the tendon [4,62]; therefore, the collective behavior of GTO’s can be approximated as linear force feedback.

The homonymous activity of GTO’s has been the subject of many studies [8,18,20,63,64,87]. However, the heteronymous activity of GTO’s in the upper limb remains poorly understood. Furthermore, the relatively few studies of heteronomous GTO activity have mostly focused on the cat hindlimb. From these studies it is known that heteronymous GTO connections do exist [41,52,88,89]. Furthermore, although heteronymous GTO feedback gains in the cat hindlimb are smaller than homonymous feedback gains [63], the cumulative effect of the heteronymous GTO connections may shape movement behavior more than homonymous GTO connections [90]. Unfortunately, to our knowledge, there are no past studies from which one could reliably infer any heteronymous GTO feedback gains of the human upper limb. Therefore, we have restricted our analysis of GTO feedback to homonymous force feedback gains. As a consequence, the closed-loop MIMO system transforming muscle activity to muscle force (represented by the inner loop in Fig 1) contains no coupling between inputs or outputs, and it is thus simply a set of uncoupled SISO systems. Therefore, previously published SISO models of GTO feedback can be used to construct the MIMO model of GTO feedback.

Van der Helm and Rozendaal evaluated GTO feedback in a single muscle by modeling the activity of GTO’s as pure force feedback [8,20]. Approximating the activity of GTO’s as pure force feedback implies that GTO activity is independent of skeletal geometry or joint dynamics; rather, it is affected only by activation dynamics and the maximum voluntary force of the associated muscle, $C_{i}$. However, van der Helm and Rozendaal also found that the muscle response oscillated excessively when the force feedback gain was too large, and that larger muscles required smaller gain values. Therefore, to avoid excessive oscillation, they took the force feedback gain to be inversely proportional to the maximum muscle force, $C_{i}$; therefore, according to this model, the feedback signal is independent of maximum muscle force. In the MIMO system, this is implemented with a diagonal matrix C containing $C_{i}$ (the same matrix C mentioned in the forward path above). In other words, the force feedback gain is equal to $kC^{-1}$, where k is a constant of proportionality. Thus, in the transformation from neural drive to GTO output, C cancels out. This results in a model in which GTO output is independent of a muscle’s maximum voluntary force, which is consistent with the assumption that, in different muscles, GTO’s are activated by a similar number of muscle fibers. Van der Helm and Rozendaal further showed that for system stability to be maintained, k must be in the range 0<k<2.7. Because the modeled GTO output is independent of skeletal geometry, joint dynamics, and maximum muscle force, this range can be applied to every muscle. In selecting a proper gain value, they noted the following tradeoff: larger gain values increased the system’s bandwidth but could also cause large oscillations at the resonant frequency. They recommended a value of 1.27, yielding a model that agrees remarkably well with in vivo measurements of muscle frequency response [83]. In summary, in our closed-loop MIMO muscle model, we modeled GTO’s as


$$\begin{array}{c} k\cdot C^{-1} \end{array}$$ (7)

Values for the maximum contractile force in C were taken from average values of a 50^th^ percentile male in the literature [13,91,92].

### 4.4 Efferent, afferent, and central delays

#### 4.4.1 Background.

The short-loop reflex can be split into three phases: afferent, central, and efferent [93]. During the afferent phase, a neural signal generated in the source muscle travels along afferent pathways to the spinal cord. The afferent latency is on the order of 10ms. However, because the travel velocity is similar for proximal and distal muscles, proximal muscles tend to have smaller afferent latencies than distal muscles. During the central phase, the neural signal is either passed directly to the motor neuron of the target muscle through a single synapse (a monosynaptic reflex arc), or else it is passed to the motor neuron of the target muscle via one or more interneurons (an oligosynaptic reflex arc). Generally, short-loop excitatory feedback is transmitted via a single synapse and inhibitory feedback is transmitted via interneurons. Neural signals take approximately 1ms to traverse each synapse, so the central latency is on the order of 1ms for excitatory feedback and 2–3ms for inhibitory feedback [35,93,94]. During the efferent phase, the neural signal travels from the spinal cord to the target muscle via motor neurons. Similar to the afferent delay, the efferent latency is on the order of 10ms, and since the travel velocity is also similar for proximal and distal muscles, proximal muscles tend to have smaller efferent latencies than distal muscles.

The round-trip delay from source muscle i to target muscle j is


$$\begin{array}{c} T_{i,j}=a_{i}+c_{i,j}+e_{j}\; \end{array}$$ (8)

where $a_{i}$ is the afferent delay from source muscle i to the spinal cord, $c_{i,j}$ is the central delay from the afferent neuron of source muscle i to the motor neuron of target muscle j, and $e_{j}$ is the efferent delay from the spinal cord to target muscle j.

#### 4.4.2 Literature values.

The literature does not contain a compiled comprehensive list of afferent, efferent, and central delays for the major superficial muscles of the arm. Nerve conduction velocities and innervation lengths have been estimated in the literature; however, reported nerve conduction velocities vary widely between studies and tend to have wide ranges even within the same study [56,82], reducing confidence in an estimation of neural delays by simply dividing the innervation length by the conduction velocity. Additionally, this method would be an indirect measurement, as nerve conduction velocities are usually estimated by dividing a delay time by nerve length. Rather, we took a more direct approach. Round-trip delays and “excess of central delays” (explained below) have been measured for many (though not all) source-target muscle pairs and are available in the literature. The reflex delay literature often considers the three heads of the deltoid as a single muscle. For measurements of neural propagation delay, this simplification is justified because the heads of the deltoid are approximately equidistant from the spinal cord, and thus delay values are expected to be very similar. This simplification results in delay measurements for 11 muscles, from which 121 source-target muscle pairs can be formed. Round-trip delay values were found in the literature for 63 of the 121 muscle pairs (see Results).

Although the details of the experimental procedure for estimating round-trip delays differed from study to study, in general they followed a similar paradigm. The source muscle was stimulated electrically or mechanically via a tendon tap, or by electrically stimulating the nerve bundle innervating the source muscle, and the time elapsed before a spike (or trough) of activity occurred in the target muscle was measured. Measurements of muscle activity were made with surface or fine-wire electromyography [35].

Central delays are difficult to measure directly. Rather, homonymous central delays were assumed to be 1ms (the approximate time for a signal to traverse a single synapse), and the amount by which heteronymous central delays exceeded the target muscle’s homonymous central delay was measured; this is called *excess central delay* [26,35,38,44]. Because facilitatory feedback generally involves only a single central synapse, the excess central delay is approximately zero for excitatory heteronymous feedback and 1–2ms for inhibitory heteronymous feedback. Thus, adding 1ms to the excess central delay yields the central delay for a given source-target muscle pair. Most articles providing information about the strength of afferent connections also provided values for excess central delay. Many PSTH articles (which observed both faciliatory and inhibitory feedback between a given muscle pair) reported excess central delay for both facilitation and inhibition; in these cases, the excess central delay value for facilitation was chosen if the feedback between a given source and target muscle was primarily faciliatory, and the excess central delay value for inhibition was chosen if the feedback was primarily inhibitory.

The central delay of each muscle is needed to estimate efferent and afferent delay times (described below). Therefore, for muscle pairs for which no excess central delay has been reported (14 out of 63 muscle pairs), we estimated the central delay as the mean of central delays for excitatory feedback (if the muscle pair exhibits excitatory feedback) or the mean of central delays for inhibitory feedback (if the muscle pair exhibits inhibitory feedback) of the muscles for which the central delay was measured.

#### 4.4.3 Estimation of afferent and efferent delays based on measurement of round-trip delays.

Knowing the round-trip delay and central delay values, we estimated the afferent and efferent delay values as follows. First, Eq. 8 was rearranged as


$$\begin{array}{c} a_{i}+e_{j}=T_{i,j}-\;c_{i,j} \end{array}$$ (9)

to isolate the two unknown variables of each muscle pair on the left. To estimate $a_{i}$ and $e_{j}$ separately for each muscle pair, we formed a system of equations of the form Ax=b using the known round-trip delay values and central delay values, with one equation per pair:


$$\left[\begin{array}{ccc} \begin{array}{ccc} 1 & 1 & 0 \end{array} & \cdots & \begin{array}{ccc} 0 & 0 & 0 \end{array}\\ \begin{array}{c} \begin{array}{ccc} 0 & 1 & 1 \end{array}\\ \vdots \\ \begin{array}{ccc} 0 & 0 & 0 \end{array} \end{array} & \begin{array}{c} \cdots \\ \ddots \\ \cdots \end{array} & \begin{array}{c} \begin{array}{ccc} 0 & 0 & 0 \end{array}\\ \vdots \\ \begin{array}{ccc} 1 & 1 & 0 \end{array} \end{array}\\ \begin{array}{ccc} 0 & 0 & 0 \end{array} & \cdots & \begin{array}{ccc} 0 & 1 & 1 \end{array} \end{array}][\begin{array}{c} a_{Delt}\\ \begin{array}{c} e_{Delt}\\ a_{Pec}\\ \vdots \end{array}\\ \begin{array}{c} e_{FCU}\\ a_{FCR}\\ e_{FCR} \end{array} \end{array}]=[\begin{array}{c} T_{Delt,Delt}-c_{Delt,Delt}\\ \begin{array}{c} T_{Pec,Delt}-c_{Pec,Delt}\\ \vdots \end{array}\\ \begin{array}{c} T_{i,j}-c_{i,j}\\ \begin{array}{c} \vdots \\ T_{FCR,FCU}-c_{FCR,FCU} \end{array}\\ T_{FCR,FCR}-c_{FCR,FCR} \end{array} \end{array}\right]$$

where A is a sparse matrix of ones and zeros, x is a vector of unknown afferent and efferent delay values, and b is a vector of values representing the sum of the afferent and efferent delay values for each pair (calculated as the difference between the total and central delays). Note that it is not necessary to have measured round-trip delay values for all muscle pairs (e.g., ECU-Delt was not measured). The afferent delay for any muscle can be estimated provided that at least one measurement was taken in which that muscle was the source muscle. Similarly, we can estimate the efferent delay for any muscle provided that at least one measurement was taken in which that muscle was the target muscle. The only muscle for which neither efferent nor afferent delay could be estimated was the Brachialis muscle (Bra), so we excluded $a_{Bra}$ and $e_{Bra}$ from x.

This system of equations is overdetermined, so A has more rows than columns and is therefore not invertible. Because of this, no solution for x exists which perfectly satisfies all conditions. However, we obtained a least-squares error solution, as described in S2 Text.

To validate this method, we calculated regression coefficients of afferent and efferent delays against innervation length [54]. The reciprocal of these regression coefficients is the average afferent and efferent conduction velocity. To validate the estimated delay values, estimated efferent and afferent conduction velocities were compared to measured values.

To estimate the afferent and efferent delay times for Bra, for which there were no measurements in the literature, we substituted the average length of nerves innervating Bra into the regression equations, yielding estimates of its afferent and efferent delays. The central delay of Bra was approximated as the mean of the excitatory or inhibitory central delays, as described previously.

## Supporting information

S1 Text *Forward Path Model* – a description of the model used as the forward path while analyzing and simulating with type Ia and GTO mechanisms. (DOCX)

S2 Text *Least-squared solution of delay times* – a description of the mathematics used to combine estimates of propagation delay parameters. (DOCX)

S3 Text *Additional tables* – a collection of tables with intermediate values generated during the steps of processing muscle connection strength values into muscle spindle feedback gain values. (DOCX)

S4 Text *Additional figures* – a collection of figures representing the placement of zeros and poles of both the open-loop and closed-loop systems, and additional frequency and step responses. (DOCX)
