# Supplementary material for: Feedback parameters for a closed-loop multiple-input multiple-output model of the upper limb
Source: PLoS Comput Biol. 2025 Jun 30;21(6):e1013183. doi: 10.1371/journal.pcbi.1013183 (PMC12244677; doi:10.1371/journal.pcbi.1013183)
Supplement: S1 Text — (DOCX) [file pcbi.1013183.s001.docx]

S1 Text

# Forward Path Model

The forward path of the model, including determination of parameter values, was described in detail by Corie et al. and Davidson et al. [1, 2], except that here both heads of the biceps were combined into one biceps muscle, and both heads of the triceps were combined into one triceps muscle. The forward path of the model consists of three submodels representing the transformations from muscle activity to muscle force, muscle force to joint torque, and joint torque to joint displacement.

# Transformation from Muscle Activity to Muscle Force

This transformation is approximated by a muscle model defined by $T_{1}T_{2}\ddot{\boldsymbol{f}}+\left( T_{1}+T_{2} \right)\dot{\boldsymbol{f}}+\boldsymbol{f}=C\boldsymbol{u}$, where the input $\boldsymbol{u}$ is a vector of muscle activity signals, the output $\boldsymbol{f}$ is a vector of muscle forces. This second-order differential equation consists of two first-order differential equations defined by time constants $T_{1}$ and $T_{2}$ representing excitation and contraction dynamics, respectively (see sections 9.0.5 and 9.3 in [3]). Matrix $C$ is a diagonal matrix of maximum muscle forces. Values for the time constants (30 and 40ms) and maximum muscle forces (Table 2 in the main article) were based on [3] and [2] respectively.

# Transformation from Muscle Force to Joint Torque

This transformation reflects the musculoskeletal geometry and is defined by $M\boldsymbol{f}=\boldsymbol{\tau}$, where the input $\boldsymbol{f}$ is a vector of muscle forces, the output $\boldsymbol{\tau}$ is a vector of joint torques, and $M$ is a matrix of moment arms (equal to the transpose of the Jacobian from muscle to joint space). Moment-arm values (Table A) were taken from OpenSim [4], with the arm set in a posture similar to that taken in the experimental studies from which gain values were derived in the main article. The row of zeros associated with SFE reflects a limitation of the moment-arm information in OpenSim (for more detail, see the explanation in the appendix of [1]).

*Table A. Matrix of muscle moment arms,* $M$ *(all values in mm)*

| **Muscle Moment Arms (mm)** | **Ant Delt** | **Lat Delt** | **Post Delt** | **Pec** | **Bi** | **Tri** | **Bra** | **Brd** | **PT** | **ECR** | **ECU** | **FCR** | **FCU** |
| --- | --- | --- | --- | --- | --- | --- | --- | --- | --- | --- | --- | --- | --- |
| **SFE** | 0 | 0 | 0 | 0 | 0 | 0 | 0 | 0 | 0 | 0 | 0 | 0 | 0 |
| **SAA** | 15.5 | -34.1 | -17.9 | 56.5 | 12.7 | 3.5 | 0 | 0 | 0 | 0 | 0 | 0 | 0 |
| **SIER** | 5.1 | 1.9 | -8.6 | 9.6 | 5.0 | -2.4 | 0 | 0 | 0 | 0 | 0 | 0 | 0 |
| **EFE** | 0 | 0 | 0 | 0 | 45.8 | -19.6 | 22.7 | 78.6 | 14.5 | 13.0 | 13.6 | 15.3 | -2.7 |
| **FPS** | 0 | 0 | 0 | 0 | -12.8 | 0 | 0 | 5.1 | 9.9 | 2.0 | 1.2 | 0.0 | -0.7 |
| **WFE** | 0 | 0 | 0 | 0 | 0 | 0 | 0 | 0 | 0 | 14.9 | 14.9 | -11.2 | -6.3 |
| **WRUD** | 0 | 0 | 0 | 0 | 0 | 0 | 0 | 0 | 0 | -7.5 | 21.8 | -17.8 | 25.1 |

# Transformation from Joint Torque to Joint Angle Displacement

The transformation from joint torque to joint rotation is represented by the second-order differential equation $I\ddot{\boldsymbol{q}}+D\dot{\boldsymbol{q}}+K\boldsymbol{q}=\boldsymbol{\tau}$, where the input $\boldsymbol{\tau}$ is a vector of joint torques, the output $\boldsymbol{q}$ is a vector of joint angles, and matrices $I$, $D$, and $K$ are the joint impedance matrices representing joint inertia, damping, and stiffness, respectively. The inertia matrix represents the inertia of the upper limb in the default posture (see Methods); its values (Table B) were computed using Matlab’s Robotics Vision and Control toolbox [5] from inertial body segment parameters of a 50^th^ percentile male taken from de Leva et al. [6]. Stiffness and damping parameter values (Table B) were estimated from a large number of past experimental studies (for details, see [2]).

*Table B. Impedance matrices* $I$*,* $D$*, and* $K$

| **Inertia (kg m^2^)** | **SFE** | **SAA** | **SIER** | **EFE** | **FPS** | **WFE** | **WRUD** |
| --- | --- | --- | --- | --- | --- | --- | --- |
| **SFE** | 0.269 | 0 | 0 | 0.076 | 0 | 0 | -0.012 |
| **SAA** | 0 | 0.196 | 0.083 | 0 | -0.002 | 0.009 | 0 |
| **SIER** | 0 | 0.083 | 0.079 | 0 | 0 | 0.011 | 0 |
| **EFE** | 0.076 | 0 | 0 | 0.076 | 0 | 0 | -0.012 |
| **FPS** | 0 | -0.002 | 0 | 0 | 0.002 | 0 | 0 |
| **WFE** | 0 | 0.009 | 0.011 | 0 | 0 | 0.003 | 0 |
| **WRUD** | -0.012 | 0 | 0 | -0.012 | 0 | 0 | 0.003 |
|  |  |  |  |  |  |  |  |
| **Damping (Nms/rad)** | **SFE** | **SAA** | **SIER** | **EFE** | **FPS** | **WFE** | **WRUD** |
| **SFE** | 0.756 | 0.184 | 0.020 | 0.187 | 0 | 0 | 0 |
| **SAA** | 0.184 | 0.383 | 0.267 | 0 | 0 | 0 | 0 |
| **SIER** | 0.020 | 0.267 | 0.524 | 0 | 0 | 0 | 0 |
| **EFE** | 0.187 | 0 | 0 | 0.607 | 0 | 0 | 0 |
| **FPS** | 0 | 0 | 0 | 0 | 0.021 | 0.000 | 0.008 |
| **WFE** | 0 | 0 | 0 | 0 | 0.000 | 0.028 | -0.003 |
| **WRUD** | 0 | 0 | 0 | 0 | 0.008 | -0.003 | 0.082 |
|  |  |  |  |  |  |  |  |
| **Stiffness (Nm/rad)** | **SFE** | **SAA** | **SIER** | **EFE** | **FPS** | **WFE** | **WRUD** |
| **SFE** | 10.80 | 2.63 | 0.28 | 2.67 | 0 | 0 | 0 |
| **SAA** | 2.63 | 5.47 | 3.82 | 0 | 0 | 0 | 0 |
| **SIER** | 0.28 | 3.82 | 7.49 | 0 | 0 | 0 | 0 |
| **EFE** | 2.67 | 0 | 0 | 8.67 | 0 | 0 | 0 |
| **FPS** | 0 | 0 | 0 | 0 | 0.76 | 0.02 | 0.29 |
| **WFE** | 0 | 0 | 0 | 0 | 0.02 | 0.99 | -0.10 |
| **WRUD** | 0 | 0 | 0 | 0 | 0.29 | -0.10 | 2.92 |

# References

1. Corie TH, Charles SK. Simulated Tremor Propagation in the Upper Limb: From Muscle Activity to Joint Displacement. J Biomech Eng-T Asme. 2019;141(8). doi: Artn 081001

10.1115/1.4043442. PubMed PMID: WOS:000473065000001.

2. Davidson AD, Charles SK. Fundamental Principles of Tremor Propagation in the Upper Limb. Ann Biomed Eng. 2017;45(4):1133-47. doi: 10.1007/s10439-016-1765-5. PubMed PMID: WOS:000398737100025.

3. Winter D. Biomechanics and Motor Control of Human Movement. 4 ed. Hoboken, New Jersey: John Wiley & Sons, Inc.; 2009.

4. Holzbaur KRS, Murray WM, Delp SL. A model of the upper extremity for simulating musculoskeletal surgery and analyzing neuromuscular control. Ann Biomed Eng. 2005;33(6):829-40. doi: 10.1007/s10439-005-3320-7. PubMed PMID: WOS:000230075100011.

5. Corke P. Robotics, Vision and Control: Fundamental Algorithms In MATLAB®. 2 ed. Cham, Switzerland: Springer International Publishing; 2017.

6. de Leva P. Adjustments to Zatsiorsky-Seluyanov's segment inertia parameters. J Biomech. 1996;29(9):1223-30. doi: 10.1016/0021-9290(95)00178-6. PubMed PMID: 8872282.
