# Supplementary material for: Feedback parameters for a closed-loop multiple-input multiple-output model of the upper limb
Source: PLoS Comput Biol. 2025 Jun 30;21(6):e1013183. doi: 10.1371/journal.pcbi.1013183 (PMC12244677; doi:10.1371/journal.pcbi.1013183)
Supplement: S2 Text — (DOCX) [file pcbi.1013183.s002.docx]

Supporting Information: S2_Text

# Least-squares solution of delay times

For the overdetermined system $A\boldsymbol{x}=\boldsymbol{b}$, a least-squares solution can be found using the Moore-Penrose pseudoinverse, $A^{\dagger}$:

$$\begin{aligned} \tilde{\boldsymbol{x}}=A^{\dagger}\boldsymbol{b} \end{aligned}$$

where $A$ is decomposed using the Singular Value Decomposition [1] as $A=U\Sigma V^{T}=\left[ \begin{matrix} U_{1} & U_{2} \end{matrix} \right]\left[ \begin{matrix} \Sigma_{1} & 0 \\ 0 & 0 \end{matrix} \right]\left[ \begin{matrix} V_{1} & V_{2} \end{matrix} \right]^{T}=U_{1}\Sigma_{1}V_{1}^{T}$, and $A^{\dagger}=V_{1}\Sigma_{1}^{-1}U_{1}^{T}$.

Note that $\tilde{\boldsymbol{x}}\mathbf{=}\left[ \tilde{a}_{Delt}, \tilde{e}_{Delt}, \tilde{a}_{Pec}, \cdots, \tilde{e}_{FCU}, \tilde{a}_{FCR}, \tilde{e}_{FCR} \right]^{T}$ has a degree of freedom: adding a constant, $d$, to each afferent delay and subtracting the same constant from each efferent delay yields a solution that is equally good (in a least-squares sense). Therefore, the values in $\tilde{\boldsymbol{x}}$ are not the final estimates. To constrain this degree of freedom, we assumed that signals travel along afferent axons at some constant velocity and efferent axons at some (possibly different) constant velocity:

$$\boldsymbol{a}\mathbf{=}\tilde{\boldsymbol{a}}+d\approx\frac{\boldsymbol{l}}{v_{a}}$$

and

$$\boldsymbol{e}\mathbf{=}\tilde{\boldsymbol{e}}-d\approx\frac{\boldsymbol{l}}{v_{e}}$$

where $\tilde{\boldsymbol{a}}=\left[ \begin{matrix} \tilde{a}_{Delt} \\ \vdots\\ \tilde{a}_{FCR} \end{matrix} \right]$, $\tilde{\boldsymbol{e}}=\left[ \begin{matrix} \tilde{e}_{Delt} \\ \vdots\\ \tilde{e}_{FCR} \end{matrix} \right]$, $\boldsymbol{l}$ is a vector of nerve lengths to each muscle [2], $v_{a}$ is the afferent conduction velocity, $v_{e}$ is the efferent conduction velocity, and $d$ is a variable that allows us to constrain the degree of freedom in the solution for $\boldsymbol{a}$. Values for $v_{a}$, $v_{e}$, and $d$ were found using the minimum squared error linear regressions $\tilde{\boldsymbol{a}}=\frac{1}{\boldsymbol{v}_{\boldsymbol{a}}}\boldsymbol{l}+d_{a}$ and $\tilde{\boldsymbol{e}}=\frac{1}{\boldsymbol{v}_{\boldsymbol{e}}}\boldsymbol{l}+d_{e}$ regressed against $\boldsymbol{l}$, where $d=\frac{d_{e}+d_{a}}{2}$. This causes the regressions for $\boldsymbol{a}$ and $\boldsymbol{e}$ to cross the vertical axis as close as possible to the origin (minimal squared error) as expected, since at an innervation length of $l=0$, the afferent and efferent conduction times should be near zero.

# References

1. Hespanha JP. Linear Systems Theory. 2 ed: Princeton University Press; 2018.

2. Kendir S, Sen T, Firat T, Leblebicioglu AG, Turker T, Tekdemir I, et al. Motor nerve lengths of twenty-seven muscles in upper extremity. Clin Anat. 2012;25(3):373-8. Epub 2011/09/08. doi: 10.1002/ca.21247. PubMed PMID: 21898604.
