# Supplementary material for: Feedback parameters for a closed-loop multiple-input multiple-output model of the upper limb
Source: PLoS Comput Biol. 2025 Jun 30;21(6):e1013183. doi: 10.1371/journal.pcbi.1013183 (PMC12244677; doi:10.1371/journal.pcbi.1013183)
Supplement: S3 Text — (DOCX) [file pcbi.1013183.s003.docx]

Supporting Information: S3_Text

# Additional Tables

*Table A. Intermediate gain precursor values after processing step 1 (dividing observed feedback between source muscles). When spinal afferents were initiated by stimulation of a nerve innervating multiple muscles (distal to the site of stimulation), the gain precursor was divided between the source muscles sharing the innervation. Columns for Delt Lat and Bra were empty and therefore excluded. Cells in gray represent homonymous reflex loops. Values of the same color were measured using the same method, and are therefore on the same scale and can be compared to each other.*

|  | | Target Muscle | | | | | | | | | | |
| --- | --- | --- | --- | --- | --- | --- | --- | --- | --- | --- | --- | --- |
|  |  | Delt Ant | Delt Post | Pec Maj | Bicep | Tricep | Brd | PT | ECR | ECU | FCR | FCU |
| Source Muscle | Delt Ant | .23 |  |  | 0.65 0.0088 |  |  |  |  |  |  |  |
|  | Delt Lat | .23 |  |  | 0.596 0.0046 |  |  |  |  |  |  |  |
|  | Delt Post | .23 | 0.5 | -0.067 | -0.012 0.019 -0.034 0  0 | 0.041 0.0107 -0.013 |  |  |  |  |  |  |
|  | Pec Maj |  | -0.052 | 0.5 | 0.032 0.009 0.065 | -0.004 0.022 -0.012 |  |  |  |  |  |  |
|  | Biceps | 0.23 | 0.008 | 0.024 | 0.5 | -0.5  -0.06 -0.047 -0.025 -0.033 |  | -0.5 | 0 0 |  | -0.24 -0.278 | -0.29 |
|  | Triceps | 0.41 | 0.005 | 0.005 | -0.51  -0.085 -0.125 0.009 -0.060 | 0.5 |  |  | -0.3 -0.348 |  | -0.21 -0.388 | -0.31 |
|  | Bra |  |  |  | -0.4129 -0.3148 | -0.033 |  |  |  |  |  |  |
|  | Brd |  | 0.0909 |  | -0.4219 -0.3148 -0.5238 | -0.5217 -0.0075 |  | -0.5484 | 0.4510 0.32 |  | -0.38 -0.0142 |  |
|  | PT |  |  |  | 0.5 0.36 | -0.2 | .0793 .5385 |  | 0.35 0.57 |  |  |  |
|  | ECR | 0.38 | 0.0909 |  | 0.3 -0.4166 | -0.2 0.4821 0.0995 |  | 0.41 0.53 | 0.38 0.5 | 0 | -0.47 |  |
|  | ECU |  | 0.0909 |  | 0.3 -0.4166 | -0.2 0.4821 0.0995 |  |  | 0.22 0.5 | 0.17 |  |  |
|  | FCR | 0 |  |  | 0.5 0.4 0.0735 | -0.2 -0.2963 | -0.3654 -0.38 0.0793 -0.1538 -0.0132 |  | -0.46 |  | 0.63 0.5 | 0.3 |
|  | FCU | 0 | 0.4091 |  | 0  0.4 0.0735 | 0 -0.2963 |  |  |  |  | 0.15 0.5 | 0.31 |

*Table B. Intermediate gain precursor values following processing step 2 (averaging within methods). Precursor values for each source/target muscle pair were averaged between studies using comparable methods. Columns for Delt Lat and Bra were empty and therefore excluded. Cells in gray represent homonymous reflex loops. Values of the same color were measured using the same method and are therefore on the same scale and can be compared to each other.*

|  | | Target Muscle | | | | | | | | | | |
| --- | --- | --- | --- | --- | --- | --- | --- | --- | --- | --- | --- | --- |
|  |  | Delt Ant | Delt Post | Pec Maj | Bicep | Tricep | Brd | PT | ECR | ECU | FCR | FCU |
| Source Muscle | Delt Ant | 0.23 |  |  | 0.65 0.0088 |  |  |  |  |  |  |  |
|  | Delt Lat | 0.23 |  |  | 0.596 0.0046 |  |  |  |  |  |  |  |
|  | Delt Post | 0.23 | 0.5 | -0.067 | -0.0068 0 | 0.0129 |  |  |  |  |  |  |
|  | Pec Maj |  | -0.052 | 0.5 | 0.0353 | 0.002 |  |  |  |  |  |  |
|  | Biceps | 0.23 | 0.008 | 0.024 | 0.5 | -0.158 -0.033 |  | -0.5 | 0 0 |  | -0.24 -0.278 | -0.29 |
|  | Triceps | 0.41 | 0.005 | 0.005 | -0.1778 -0.060 | 0.5 |  |  | -0.3 -0.348 |  | -0.21 -0.388 | -0.31 |
|  | Bra |  |  |  | -0.3639 | -0.033 |  |  |  |  |  |  |
|  | Brd |  | 0.0909 |  | -0.4202 | -0.5217 -0.0075 |  | -0.5484 | 0.3855 |  | -0.38 -0.0142 |  |
|  | PT |  |  |  | 0.43 | -0.2 | 0.3089 |  | 0.46 |  | -0.125 |  |
|  | ECR | 0.38 | 0.0909 |  | -0.0583 | 0.1411 0.0995 |  | 0.47 | 0.38 0.5 | 0 | -0.47 |  |
|  | ECU |  | 0.0909 |  | -0.0583 | 0.1411 0.0996 |  |  | 0.22 0.5 | 0.17 |  |  |
|  | FCR | 0 |  |  | 0.45 0.0735 | -0.2482 | -0.2050 -0.0132 |  | -0.46 |  | 0.63 0.5 | 0.3 |
|  | FCU | 0 | 0.4091 |  | 0.2 0.0735 | -0.14815 |  |  |  |  | 0.15 0.5 | 0.31 |

*Table C. Intermediate gain precursor values following processing step 3 (values from each method were transformed to be on the same scale). Values derived from different methods were placed on the same scale using linear regressions. Columns for Delt Lat and Bra were empty and therefore excluded. Cells in gray represent homonymous reflex loops. Values of the same color were measured using the same method.*

|  | | Target Muscle | | | | | | | | | | |
| --- | --- | --- | --- | --- | --- | --- | --- | --- | --- | --- | --- | --- |
|  |  | Delt Ant | Delt Post | Pec Maj | Bicep | Tricep | Brd | PT | ECR | ECU | FCR | FCU |
| Source Muscle | Delt Ant | 0.23 |  |  | 0.65 0.0613 |  |  |  |  |  |  |  |
|  | Delt Lat | 0.23 |  |  | 0.596 0.0320 |  |  |  |  |  |  |  |
|  | Delt Post | 0.23 | 0.5 | -0.067 | -0.0068 0 | 0.0129 |  |  |  |  |  |  |
|  | Pec Maj |  | -0.052 | 0.5 | 0.0353 | 0.002 |  |  |  |  |  |  |
|  | Biceps | 0.23 | 0.008 | 0.024 | 0.5 | -0.158 -0.2299 |  | -0.5 | 0 0 |  | -0.24 -0.2012 | -0.29 |
|  | Triceps | 0.41 | 0.005 | 0.005 | -0.1778 -0.4179 | 0.5 |  |  | -0.3 -0.2519 |  | -0.21 -0.2809 | -0.31 |
|  | Bra |  |  |  | -0.3639 | -0.2299 |  |  |  |  |  |  |
|  | Brd |  | 0.0909 |  | -0.4202 | -0.5217 -0.0522 |  | -0.5484 | 0.3855 |  | -0.38 -0.0989 |  |
|  | PT |  |  |  | 0.43 | -0.2 | 0.3089 |  | 0.46 |  |  |  |
|  | ECR | 0.38 | 0.0909 |  | -0.0583 | 0.1411 0.2466 |  | 0.47 | 1.2381 1.2381 | 0 | -0.47 |  |
|  | ECU |  | 0.0909 |  | -0.0583 | 0.1411 0.2466 |  |  | 1.2381 1.2381 | 0.9567 |  |  |
|  | FCR | 0 |  |  | 0.45 0.1820 | -0.2482 | -0.2050 -0.0919 |  | -0.46 |  | 1.2381 1.2381 | 0.5896 |
|  | FCU | 0 | 0.4091 |  | 0.2 0.1820 | -0.14815 |  |  |  |  | 1.2381 1.2381 | 2.5587 |

*Table D. Values from each measurement technique were scaled to be on equal footing using a linear regression. The multiplier indicates the scaling factor applied to values in Table C3. Parameter N indicates the number of data points sharing both colors, and R is the regression coefficient indicating agreement between measurements from different techniques, along with the associated p-value. Since multiple points are required to obtain a meaningful correlation coefficient and p-value, no R or p-values are listed for rows with N = 1.*

|  | **Multiplier** | ***N*** | ***R*** | ***p*** |
| --- | --- | --- | --- | --- |
| Pink to Red | 0.72 | 4 | 0.98 | 0.0043 |
| Light Blue to Red | 2.48 | 4 | 0.82 | 0.092 |
| Green to Red | 6.97 | 6 | 0.80 | 0.031 |
| Orange to Light Blue | 8.25 | 1 | NA | NA |
| Yellow to Light Blue | 1.97 | 1 | NA | NA |
| Dark Blue to Light Blue | 3.26 | 1 | NA | NA |
| Purple to Light Blue | 5.63 | 1 | NA | NA |
