# Supplementary material for: Feedback parameters for a closed-loop multiple-input multiple-output model of the upper limb
Source: PLoS Comput Biol. 2025 Jun 30;21(6):e1013183. doi: 10.1371/journal.pcbi.1013183 (PMC12244677; doi:10.1371/journal.pcbi.1013183)
Supplement: S4 Text — (DOCX) [file pcbi.1013183.s004.docx]

Supporting Information: S4_Text

# Additional Figures


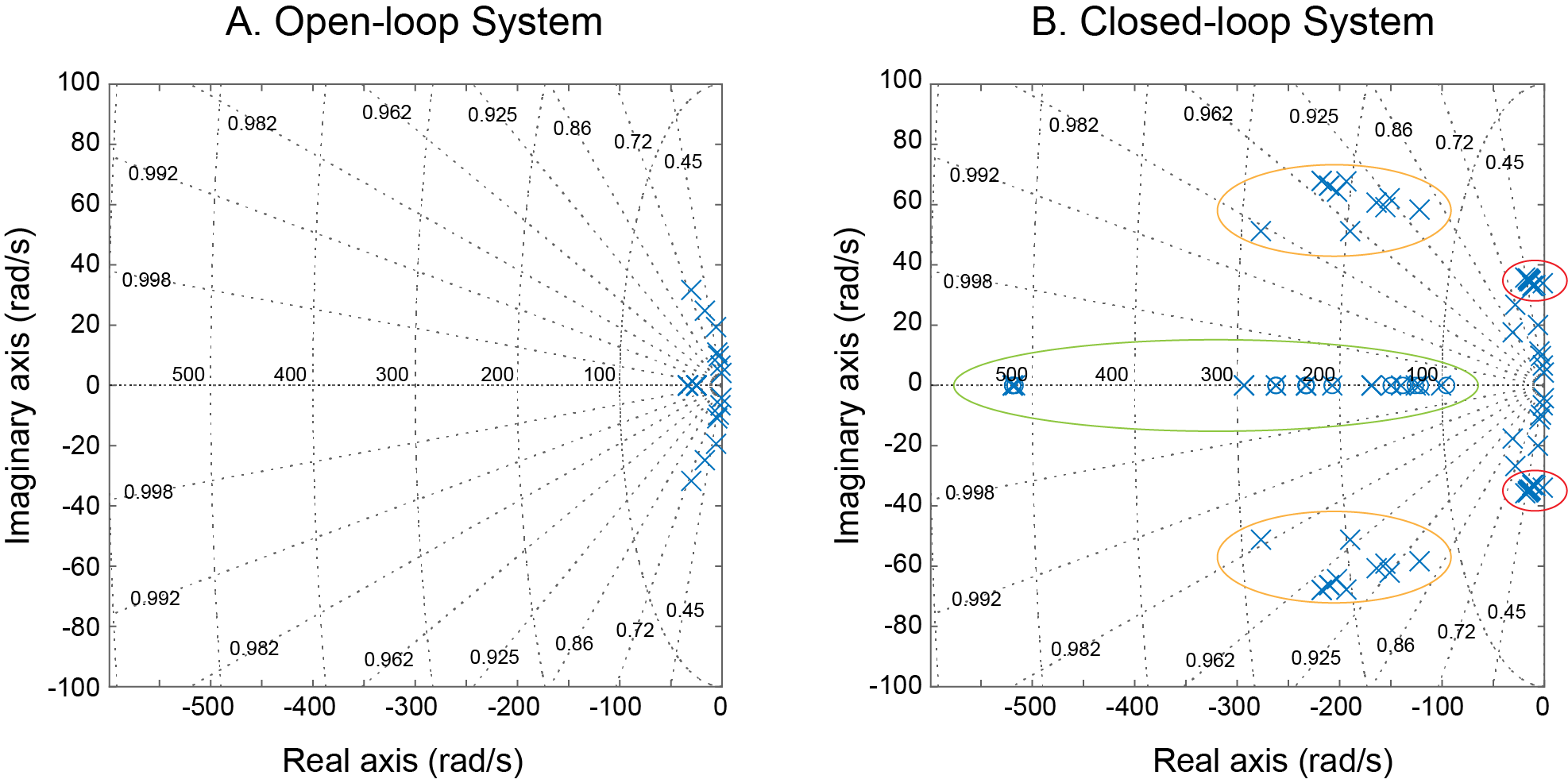


*Fig A.* *Pole-zero plots of open-loop system (A) and closed-loop system (B). Including afferent feedback adds three clusters of poles and zeros, circled in green, yellow, and red.*


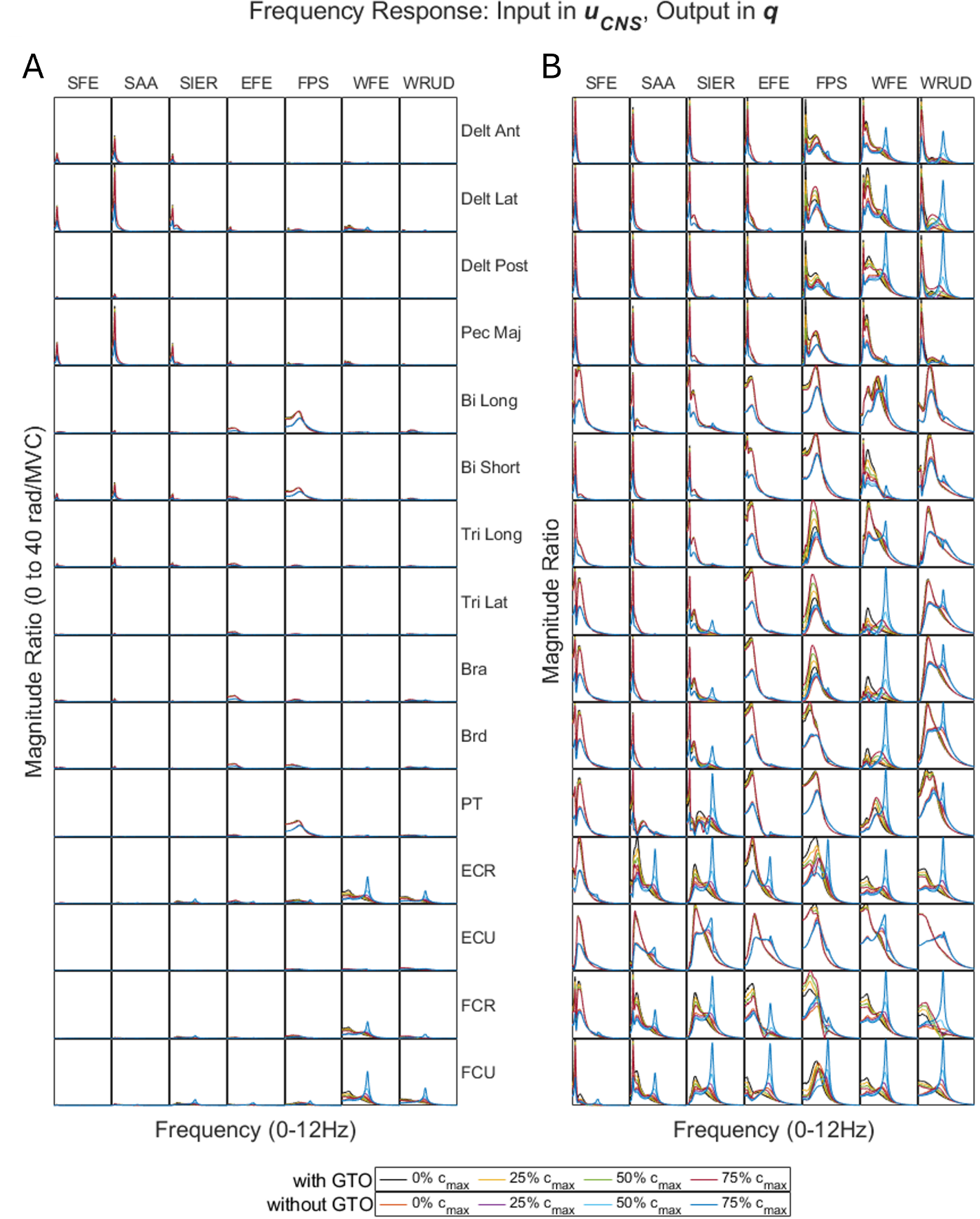


*Fig B. A) Frequency response of joint angle (*$\boldsymbol{q}$*) due to unit step inputs in descending neural drive (*$\boldsymbol{u}_{CNS}$*). The units of the magnitude ratios are radians divided by the units of descending neural drive (MVC). B) Same as A, but zoomed in, such that each subplot has its own distinct magnitude ratio range (along the vertical axis).*


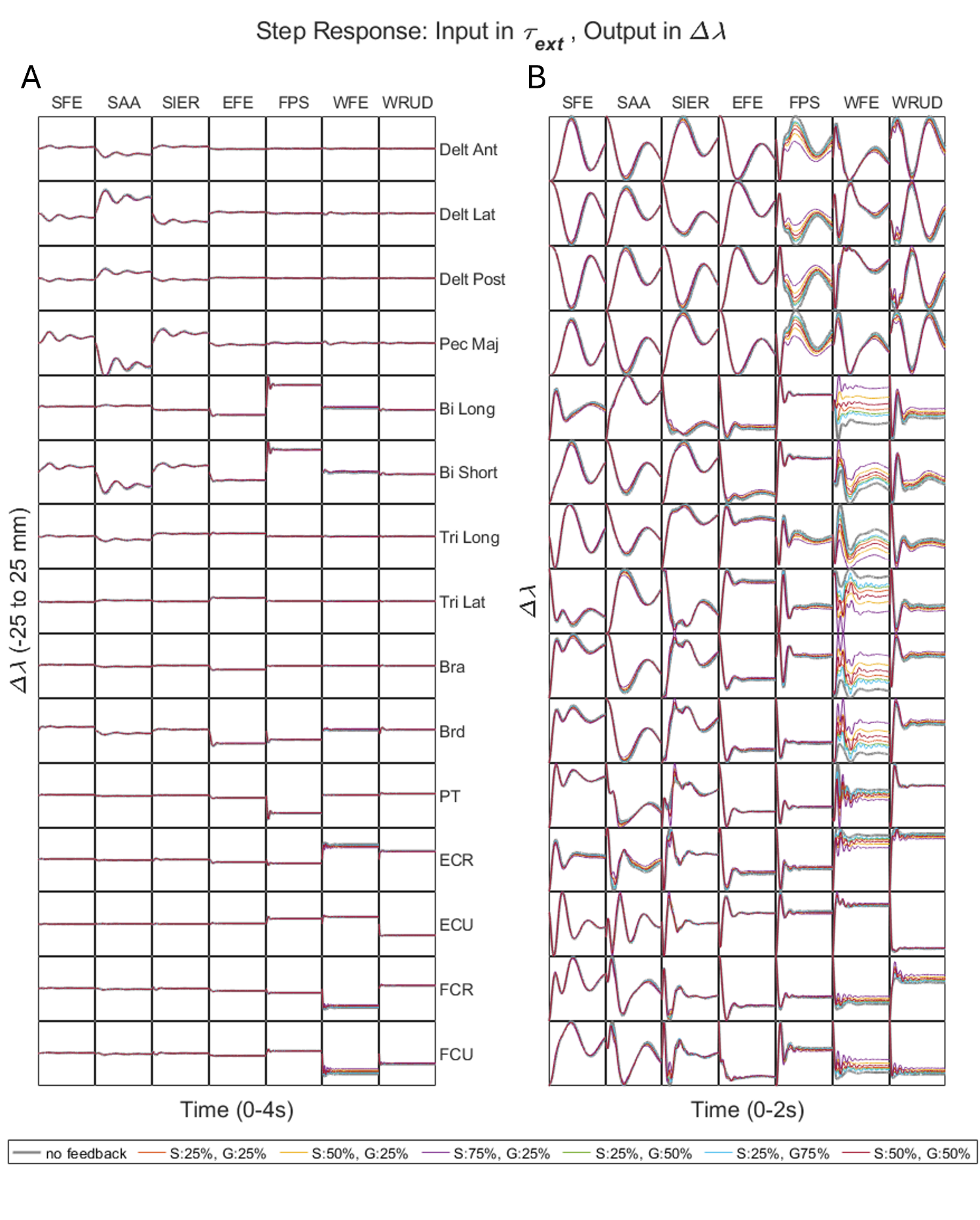


*Fig C. A) Step responses from external joint torque to change in muscle length (i.e. response in* $\boldsymbol{\Delta\lambda}$ *due to step in* $\boldsymbol{\tau}_{ext}$*), with equal time and change-in-muscle-length ranges (on the horizontal and vertical axes, respectively) for all subplots. B) Same as A, but zoomed in, such that each subplot has its own distinct change-in-muscle-length range (along the vertical axis).*
